# Novel RU486 (mifepristone) analogues with increased activity against Venezuelan Equine Encephalitis Virus but reduced progesterone receptor antagonistic activity

**DOI:** 10.1038/s41598-019-38671-y

**Published:** 2019-02-22

**Authors:** Aaron DeBono, David R. Thomas, Lindsay Lundberg, Chelsea Pinkham, Ying Cao, J. Dinny Graham, Christine L. Clarke, Kylie M. Wagstaff, Sharon Shechter, Kylene Kehn-Hall, David A. Jans

**Affiliations:** 1 Shechter Computational Solutions, Andover, MA USA; 2 Nuclear Signaling Laboratory, Department of Biochemistry and Molecular Biology School of Biomedical Sciences, Monash University, Melbourne, Australia; 3 National Center for Biodefense and Infectious Diseases, School of Systems Biology, George Mason University, Manassas, VA USA; 4 Medicinal Chemistry, Monash Institute of Pharmaceutical Sciences, Monash University, Parkville, Victoria 3052 Australia; 5 Centre for Cancer Research, The Westmead Institute for Medical Research, Westmead, NSW Australia

## Abstract

There are currently no therapeutics to treat infection with the alphavirus Venezuelan equine encephalitis virus (VEEV), which causes flu-like symptoms leading to neurological symptoms in up to 14% of cases. Large outbreaks of VEEV can result in 10,000 s of human cases and mass equine death. We previously showed that mifepristone (RU486) has anti-VEEV activity (EC_50_ = 20 μM) and only limited cytotoxicity (CC_50_ > 100 μM), but a limitation in its use is its abortifacient activity resulting from its ability to antagonize the progesterone receptor (PR). Here we generate a suite of new mifepristone analogues with enhanced antiviral properties, succeeding in achieving >11-fold improvement in anti-VEEV activity with no detectable increase in toxicity. Importantly, we were able to derive a lead compound with an EC_50_ of 7.2 µM and no detectable PR antagonism activity. Finally, based on our SAR analysis we propose avenues for the further development of these analogues as safe and effective anti-VEEV agents.

## Introduction

Venezuelan equine encephalitis virus (VEEV) is an emerging pathogenic *alphavirus*, family *Togaviradae*, which induces a febrile illness and encephalitis in humans with around 14% of cases develop neurological disease^1,2^. VEEV has been the cause of outbreaks involving tens to hundreds of thousands of equine and human cases spreading over large geographical areas and lasting years. The 1969 outbreak in Central America, Mexico, and Texas, for example, lasted over 3 years, while the 1995 epidemic led to up to 100,000 human cases^1,3–5^. There is currently no available FDA-approved treatment, and the only vaccine is restricted to at-risk personnel and animals, due to its limited efficacy and high rates of side-effects.

We previously identified mifepristone (RU486) in a high throughput screen for agents able to inhibit HIV Integrase binding to the importin α/β1 (Impα/β1) cellular nuclear import proteins^6^, disrupting its nuclear trafficking and subsequently demonstrating anti-HIV activity^7,8^. Interestingly, we were also able to show that mifepristone had anti-VEEV activity^9^, as well as being able to reduce nuclear accumulation of VEEV capsid protein (CP) in infected cells; since CP nuclear import is a key contributor to pathogenicity in VEEV- infected cells^10–14^, mifepristone’s ability to reduce CP accumulation has been postulated to account at least in part for its anti-VEEV activity.

Although not approved for VEEV infection, mifepristone is FDA-approved for other indications, meaning that it has an established safety profile^15^, and hence is an excellent starting point for further development. Its abortifacient properties, however, limit its use as an antiviral^16,17^. Mifepristone’s abortifacient activity stems from its ability to antagonize the progesterone receptor (PR) by directly competing with progesterone for binding to the receptor^18,19^.

This study explores the synthesis of a series of mifepristone-like derivatives with the aim of increasing anti-viral activity while maintaining low toxicity. We were able to improve on mifepristone’s activity by >11-fold, with no detectable increase in toxicity. Importantly, we were able to strongly reduce the PR antagonism activity associated with mifepristone, with lead compound **50** lacking detectable PR antagonism, as well as being able to inhibit the nuclear import of CP, hypothesized to be involved in mifepristone’s mode of action. This work provides important new insights into the structural elements of these mifepristone-like derivatives and their respective contributions to anti-VEEV activity, PR antagonism, and CP nuclear import modulation.

## Results

**Figure 1:**
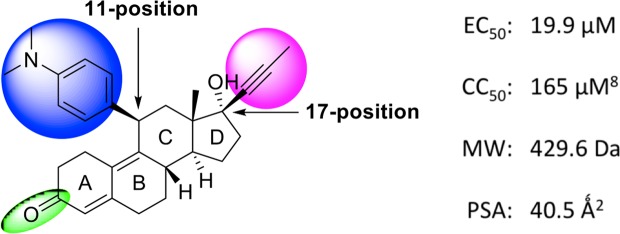
Structure of hit compound 1 (mifepristone). SAR targets at the 11- and 17-positions, and carbonyl group, are highlighted.

Mifepristone (**1**) is a FDA-approved drug, possessing a low polar surface area of 40.5 Ǻ^2^ indicating reasonable CNS penetration^20,21^. The steroid chemotype (Fig. 1) is an attractive starting point since the compound demonstrates versatility with respect to functional group manipulation at the 11- and 17-positions on the steroidal backbone, which could provide valuable SAR. We set out here to generate a series of new mifepristone analogues, to explore key functional moieties with respect to mifepristone’s antiviral activity, as measured by its ability to inhibit VEEV infection (indicated by EC_50_; see below), with other assays performed in parallel on representative analogues of interest.

### Synthesis of mifepristone analogues

Analogues were generally prepared in a 4-step procedure, beginning with the starting material estradiene dione-3-keta (**2**). The first step was the epoxidation of **2**, followed by Grignard addition at the 11-position, lithiation reaction at the 17-position, and diketal hydrolysis and 5*β*-hydroxy elimination (Supp. Fig. S1). The synthesis of specific compounds is explained in detail in the Methods and Supplemental Data Sections.

### Activities of mifepristone analogues

Compounds were initially assessed for cytotoxicity (CC_50_) and the ability to inhibit VEEV replication (EC_50_). The CC_50_ of each compound was measured by serial dilution on Vero cells. Cytotoxicity was assessed after 24 h treatment using Promega’s CellGlo Cell Viability assay normalized to DMSO treated controls. None of the mifepristone analogues indicated toxicity, with CC_50_ values > 100 uM. In addition to confirming the low toxicity of the analogues in short term treatments, this validates the EC_50_ results as being a result of viral inhibition, rather than decreasing viral replication due to cell death.

The EC_50_ of each compound was determined using a VEEV TC-83 virus expressing a luciferase reporter gene (TC-83-luc) under control of the VEEV subgenomic promoter^22^; this system is widely used as it functions independently of host gene expression which is inhibited by VEEV CP^9–11,23^. Serial dilutions of each compound were used to pre-treat Vero cells for 2 h prior to infection, after which cells were washed and the viral inoculum replaced with medium containing compound, with viral replication measured 16 hours post-infection using Promega’s BrightGlo Luciferase assay.

### SAR at the 17-position

Parent compound **1** showed an EC_50_ of 19.9 μM and a CC_50_ of 165 µM, equating to a selectivity index (SI; CC_50_/EC_50_) of 8.3. The first area targeted for modification was the 17-position, occupied by a propyne group in mifepristone (Table 1). 14 analogues were produced differing from **1** at only this region. Replacing the propyne group with a propene (**7**) resulted in a modest reduction in activity (EC_50_ = 25.3 µM) from **1**, while the addition of a hydroxy group to either the propyne or propene group abolished any detectable antiviral activity (ie, **11** and **13**). Replacement of the propyne with a cyano-group was not tolerated, with no inhibition of viral replication detected from compounds **26** and **27**.

**Table 1:** Summary for Results of SAR at the 17-position of mifepristone.

Cmpd	Position 17			EC_50_, µM^a^	CC_50_, µM^a^	SI
		R^3^	R^4^			
**34**		OH	CCSi(Et)_3_	4.25 ± 0.4	>100	>23.52
**39**		OH	CC-4-OMeC_6_H_4_	5.23 ± 1.0	>100	>19.13
**38**		OH	CCC_6_H_5_	8.46 ± 1.7	>100	>11.82
**33**		OH	CCSi(Me)_3_	8.49 ± 1.4	>100	>11.78
**21**		COCH_3_	OCOCH_3_	11.53 ± 7.0	>100	>8.67
**35**		OH	CCSi(^*i*^Pr)_3_	16.34 ± 4.7	>100	>6.12
**9**		OH	CCCF_3_	17.8 ± 1.7	>100	>5.62
**29**		CF_3_	OH	19.6 ± 2.8	>100	>5.1
**1 (Mifepristone)**		OH	CCCH_3_	19.94 ± 2.7	165	8.3
**7**		OH	CHCHCH_3_	25.33 ± 6.2	>100	>3.95
**25**		COCH_3_	OH	43.7 ± 17.8	>100	>2.29
**11**		OH	CCCH_2_OH	ND	>100	ND
**13**		OH	CHCHCH_2_OH	ND	>100	ND
**26**		H	CN	ND	>100	ND
**27**		CN	H	ND	>100	ND
**28**		OH	CF_3_	ND	>100	ND

^a^Mean ± SD, n ≥ 2; CC_50_, concentration yielding 50% cytotoxicity; EC_50,_ concentration yielding 50% inhibition of viral replication; SI, selectivity index (CC_50_/EC_50_); ND, 50% inhibition not reached at 100 µM.

**25**, containing only a single acetyl group at the 17-position possessed significantly reduced activity compared to **1** (EC_50_ = 43.7 µM), while **21**, containing an additional acetyl group, had improved activity (EC_50_ = 11.5 µM), suggesting the bulk provided by mifepristone’s propyne group may be important. Compounds **28** and **29** both possess a trifluoromethyl moiety instead of a propyne, but we were unable to determine their steric conformations (see Materials and Methods). While **29** possessed an EC_50_ comparable to **1**, **28** lacked detectable anti-VEEV activity. Overall, these results confirm the essential nature of the 17-position for anti-VEEV activity, and the preference for larger moieties.

As larger moieties seemed to be preferred, additional compounds were produced building on the propyne of **1**. The addition of a trifluoromethyl group (**9**) did not significantly alter the activity of the analogue from **1**. However, the addition of the larger TMS (**33**), TES (**34**), and TIPS (**35**) groups was favored, reducing the compounds EC_50_s to 8.5, 4.3, and 16.3 µM, respectively. Replacing the silyl groups with a terminal aromatic group also resulted in increased activity compared to **1**, with compounds **38** and **39** possessing EC_50_s of 8.5 and 5.2 µM, respectively. Collectively, these results indicate that larger hydrophobic groups at the 17-position are associated with greater anti-VEEV activity, and that terminal hydrogen bond donors are not tolerated.

### SAR at the 11-position

The N,N−dimethylamino moiety at the 11-position of **1** is strongly associated with its PR antagonism, so we looked to examine the activity of analogues lacking this domain. However, removing a single methyl group from **1** to create **6** also eliminated any detectable antiviral activity. Due to its improved activity and ease of production, compound **33** (EC_50_ = 8.5 µM) and its dioxolane analogue **30** (EC_50_ = 11.1 µM; both containing TMS at the 17-position) were instead used as backbones to test a wider range of 11-position substitutions (Table 2). Pleasingly, removing the N,N−dimethylamino moiety from **33** to produce **50** (EC_50_ = 7.2 µM) did not significantly impair its anti-VEEV activity, indicating this domain is not a requirement for activity. From **33**, the replacement of the two amino groups with either a single hydroxyl (**52**) or methoxy (**51**) group reduced anti-VEEV activity to levels comparable to **1** (EC_50_s of 21.8 and 22.5 µM, respectively). Interestingly, the addition of the methoxy group on a dioxolane backbone (**46**) resulted in a slight increase in activity, to an EC_50_ of 7 µM. Moving the N,N−dimethylamino from the 4-position to the 3-position was also seen to increase the anti-VEEV activity in both dioxolane and ketone backbones (EC_50_s of 7.5 and 6 µM for **49** and **54**, respectively). The largest improvements, however, were achieved in compounds with terminal aromatic groups, such as phenyl, OTHP, and biphenyl moieties (ie, compounds **45, 47,** and **48** with EC_50_s of 2.6, 2.9, and 3.2 µM, respectively). While terminal aromatic groups were less beneficial in the ketone backbone, a modest improvement was achieved with the addition of the biphenyl group (ie, compound **53** with an EC_50_ of 5 µM). This indicates that, in combination with TMS at the 17-position, bulky hydrophobic groups at the 11-position are not only well-tolerated, but can lead to enhanced activity.

**Table 2:** Summary of results for SAR at the 11-position of **30** and **33**.

Cmpd	Position 11			EC_50_, µM^a^	CC_50_, µM^a^	SI
		R^1^	R^2^			
**Ketone analogues**						
**53**		Ph	H	5.0 ± 0.7	>100	>20.1
**54**		H	NMe_2_	6.0 ± 1.1	>100	>16.7
**50**		H	H	7.2 ± 1.2	>100	>13.9
**33**		NMe_2_	H	8.5 ± 1.4	>100	>11.8
**52**		OH	H	21.9 ± 5.6	>100	>4.6
**51**		OMe	H	22.5 ± 2.4	>100	>4.5

**45**		H	H	2.6 ± 0.4	>100	>38.7
**47**		OTHP	H	2.9 ± 0.4	>100	>34.3
**48**		Ph	H	3.2 ± 0.6	>100	>31.1
**46**		OMe	H	7.0 ± 0.9	>100	>14.2
**49**		H	NMe_2_	7.5 ± 1.2	>100	>13.4
**30**		NMe_2_	H	11.1 ± 1.9	>100	>9.0

^a^Mean ± SD, n ≥ 2; CC_50_, concentration yielding 50% cytotoxicity; EC_50,_ concentration yielding 50% inhibition of viral replication; SI, selectivity index (CC_50_/EC_50_).

### SAR of the Ketone/dioxolane backbone

Finally, 10 sets of ketone/dioxolane homologues were produced to examine the role of the carbonyl group in anti-VEEV activity (Table 3). Interestingly, the effect of substituting it for a dioxolane group was dependent on the moieties present at the 11- and 17-positions. In pairs lacking a terminal hydrophobic ring at either position (pairs ***1***, ***2***, ***3***, ***4***, and ***5***), the ketone was either more effective against VEEV than the dioxolane version, or the two were equivalent. Conversely, where a terminal phenyl ring was present (pairs ***6***, ***7***, and ***8***), anti-VEEV activities were either improved by substituting the ketone for a dioxolane moiety or the two were equivalent. The exceptions to this observation were pairs ***9*** and ***10***, which lacked a terminal ring, but were still more active in the dioxolane backbone. What these groups had in common was a terminal methoxy group at the 11-position (pair ***10***) or 17-position (pair ***9***). This suggests that charge/polarity over the entire molecule modulates antiviral activity.

**Table 3:** Summary of results for SAR of ketone/dioxolane homologues.

Pair	Cmpd	Structure	EC_50_, µM^a^ CC_50_, µM^a^ SI	Cmpd	Structure	EC_50_, µM^a^ CC_50_, µM^a^ SI	P3 ratio
	Ketone homologue			Dioxolane homologue			
***1***	**9**		17.8 ± 1.7 >100 >5.6	**8**		27.6 ± 5.1 >100 >3.6	0.64
***2***	**35**		16.3 ± 4.7 >100 >6.1	**32**		22.0 ± 6.1 >100 >4.5	0.74
***3***	**33**		8.5 ± 1.4 >100 >11.8	**30**		11.1 ± 1.9 >100 >9.0	0.77
***4***	**54**		6.0 ± 1.1 >100 >16.7	**49**		7.5 ± 1.2 >100 >13.4	0.80
***5***	**34**		4.3 ± 0.4 >100 >23.5	**31**		4.7 ± 0.5 >100 >21.5	0.91
***6***	**38**		8.5 ± 1.7 >100 >11.8	**36**		7.2 ± 2.3 >100 >14.0	1.18
***7***	**53**		5.0 ± 0.7 >100 >20.1	**48**		3.2 ± 0.6 >100 >31.1	1.56
***8***	**50**		7.2 ± 1.2 >100 >13.9	**45**		2.6 ± 0.4 >100 >38.7	2.77
***9***	**39**		5.2 ± 1.0 >100 >19.1	**37**		1.7 ± 0.6 >100 >60.3	3.06
***10***	**51**		22.5 ± 2.4 >100 >4.5	**46**		7.0 ± 0.9 >100 >14.2	3.21

^a^Mean ± SD, n ≥ 2; CC_50_, concentration yielding 50% cytotoxicity; EC_50,_ concentration yielding 50% inhibition of viral replication; SI, selectivity index (CC_50_/EC_50_); P3 ratio, EC_**50**_ of ketone analogue/EC_**50**_ of dioxolane analogue: ratios >1 and <1 indicate the dioxolane or ketone analogue was more active, respectively.

### Optimised analogues reduce infectious virus production

To complement the insights into the ability of the compounds to inhibit viral replication provided by the luciferase assay, selected compounds with low EC_50_s were assessed for effects on infectious virus production using a plaque assay. Briefly, cells were pretreated with DMSO alone or supplemented with 10 µM of compound for 2 h before infection with VEEV TC83, and a further 16 h post treatment (Table 4). Although the luciferase and plaque assays assess different parameters with respect to the VEEV lifecycle, the plaque assay results generally correlated well with the luciferase assay, with compounds decreasing viral titers by up to 99%. Of note, compounds **36–39**, which possessed terminal aromatic groups at the 17-position, reduced viral titre by only c. 50–70%, whereas other compounds with similar EC_50_s consistently inhibited viral production by >80%, the implication being that the analogues may affect multiple aspects of the VEEV lifecycle to differing extents; while viral replication is inhibited by compounds with bulky hydrophobic rings at either the 11- or 17-position, it seems these moieties are only favorable for inhibiting infectious viral production when at the 11-position.

**Table 4:** Summary of results for viral titre reduction of selected compounds.

Cmpd	EC_50_, µM^a^	Titre reduction (%)^a^
37	1.7 ± 0.6	67.1 ± 12.2
45	2.6 ± 0.4	95.4 ± 4.1
47	2.9 ± 0.4	98.8 ± 0.5
48	3.2 ± 0.6	97.1 ± 2.1
34	4.3 ± 0.4	95.8 ± 1.9
31	4.7 ± 0.5	93.9 ± 3.2
53	5 ± 0.7	91.1 ± 5.4
39	5.2 ± 1	47.4 ± 21.4
54	6 ± 1.1	94.1 ± 7.1
46	7 ± 0.9	93.6 ± 3.1
36	7.2 ± 2.3	53.9 ± 18.8
50	7.2 ± 1.2	86.1 ± 7.5
49	7.5 ± 1.2	67.6 ± 21.0
38	8.5 ± 1.7	69.7 ± 15.4
30	11.1 ± 1.9	88.4 ± 4.8
1	19.9 ± 2.7	93.7 ± 19.6
52	21.9 ± 5.6	84.0 ± 22.3
32	22 ± 6.1	89.7 ± 4.6

^a^Mean ± SD (n ≥ 2); EC_50_, concentration yielding 50% inhibition of viral infection.

### Mifepristone and its analogues can reduce VEEV capsid nuclear import

Apart from its key role in virion structure, the VEEV CP functions to inhibit the host antiviral response^12–14,24,25^, in part by interacting with the host cell nuclear import and export machinery (the Impα/β1 heterodimer and CRM1, respectively); when these interactions are prevented, the pathogenicity of VEEV is greatly attenuated^9–11,23^. Significantly, **1** has been found to inhibit the nuclear accumulation of VEEV CP in infected cells, which is thought to be integral to its anti-VEEV activity^9^.

**Figure 2:**
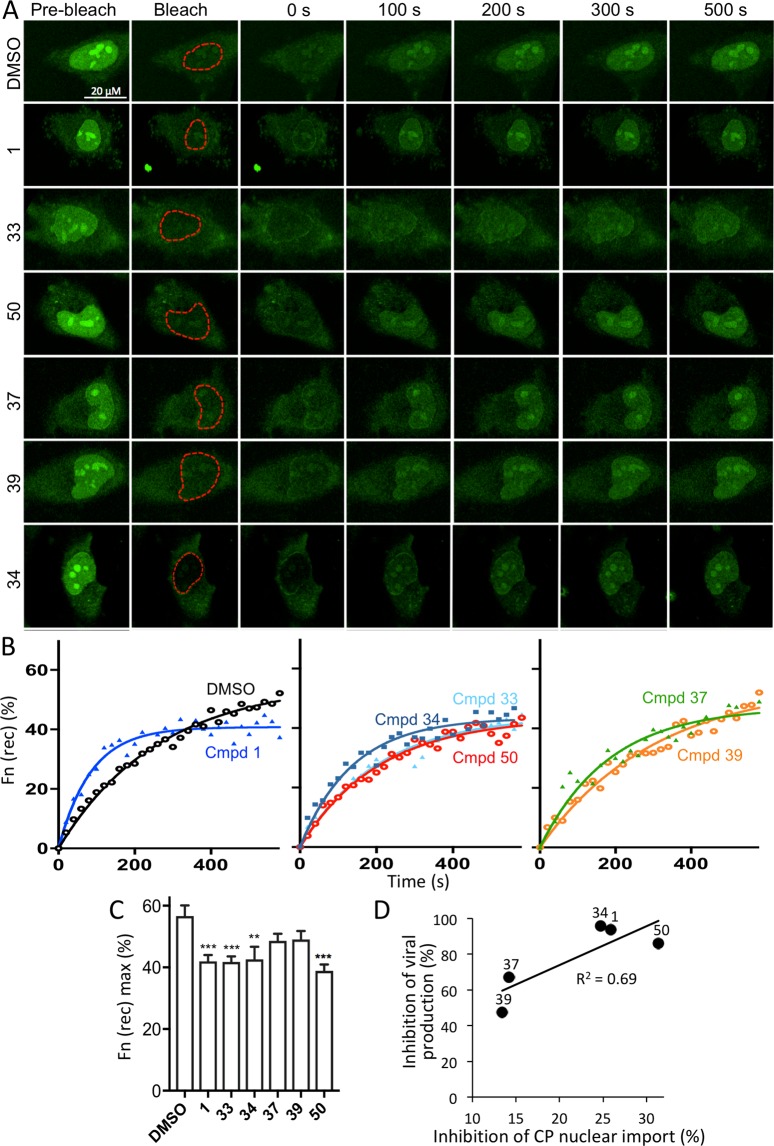
Lead compounds inhibit nuclear import of capsid in live cells. HeLa cells transfected to express a GFP-capsid fusion protein were treated with 200 µM compound or DMSO for 2 h prior to FRAP analysis as per Materials and Methods. The nuclear region (outlined in red) was photo-bleached and fluorescence recovery in this region was measured every 20 s for 580 s. (**A**) Representative CLSM images of cells prebleach, immediately following bleaching (0 s), and at the indicated times during recovery. (**B**) Images from the cells shown in A were analysed for the recovery of nuclear fluorescence. Pooled analysis of curves such as those shown in B were analysed to determine the maximal fractional recovery (Fn(rec) max, **C**). Data represents the mean ± SEM (n ≥ 17). **p < 0.01 and ***p < 0.001. (**D**) The ability of compounds to inhibit the nuclear import of CP plotted against their reduction of viral titre. Linear regression was fitted to the data (y = 2.18x + 30.32, R^2^ = 0.69), with individual compounds indicated.

To confirm that the analogues could inhibit CP nuclear import, fluorescence recovery after photobleaching (FRAP) experiments were performed. Briefly, HeLa cells were transfected to express a CP-GFP fusion protein and imaged live 24 h later by confocal laser scanning microscopy (CLSM). Fresh media containing DMSO with or without compound was added, prior to FRAP analysis 2 h later, which was performed, as previously^10,11^, by irreversibly bleaching the nuclear region (indicated by the dashed red lines in Fig. 2A), and imaging the cells every 20 s up to almost 10 min (Fig. 2A,B). Recovery of nuclear fluorescence (Fn(rec); see Materials and Methods) can only occur through transport from the cytoplasm to the nucleus, enabling nuclear import kinetics to be quantified^26–28^.

To assess the role of different structural domains in modulating activity, analogues **33, 34, 37, 39**, and **50**, all of which have EC_50_s below 10 µM, in addition to **1**, were tested. Comparison of **33** and **50** enables conclusions to be drawn as to the importance of the N,N−dimethylamino moiety at the 11-position, while that for **37** and **39** enables a comparison between the dioxolane and ketone moiety at the 3-position; **33** and **39**, in fact, only differ from **1** in terms of the 17-position moieties (possessing either trimethysilylacetylene or 4-methoxyphenylacetylene, respectively (see Table 1). Analysis of these compounds enabled us to assess how changes to the lead core **1** impact inhibition of nuclear import of CP.

**1**, **33, 34**, and **50** were all able to inhibit the nuclear import of CP-GFP significantly (p < 0.01) by c. 30% (Fig. 2B,C). **37** and **39**, which were only moderately active in plaque assays, did not significantly alter CP nuclear import dynamics, their reduced ability to inhibit CP nuclear transport thus correlating with their relative ineffectiveness in inhibiting virus production (Fig. 2D). Overall, there was a clear correlation between ability to inhibit CP nuclear import, and the ability to inhibit VEEV virus production (Fig. 2D), consistent with the idea that **1** and its analogues decrease VEEV pathogenicity in part by limiting CP nuclear transport. While this activity is reduced in compounds with bulky aromatic groups at the 17-position (eg. compounds **37** and **39**), matching the plaque assay data, they still possess low EC_50_s, indicating the presence of additional anti-VEEV mechanism(s) acting on viral replication.

### Mifepristone analogues with reduced progesterone receptor antagonism

Upon progesterone binding, the progesterone receptor (PR) translocates into the nucleus to recruit transcriptional co-regulators and impact the transcription of specific target genes. **1** is known to act by competing with progesterone, as well as other PR agonists, for binding to the PR, thereby inhibiting PR signaling^18,29^.

**Figure 3:**
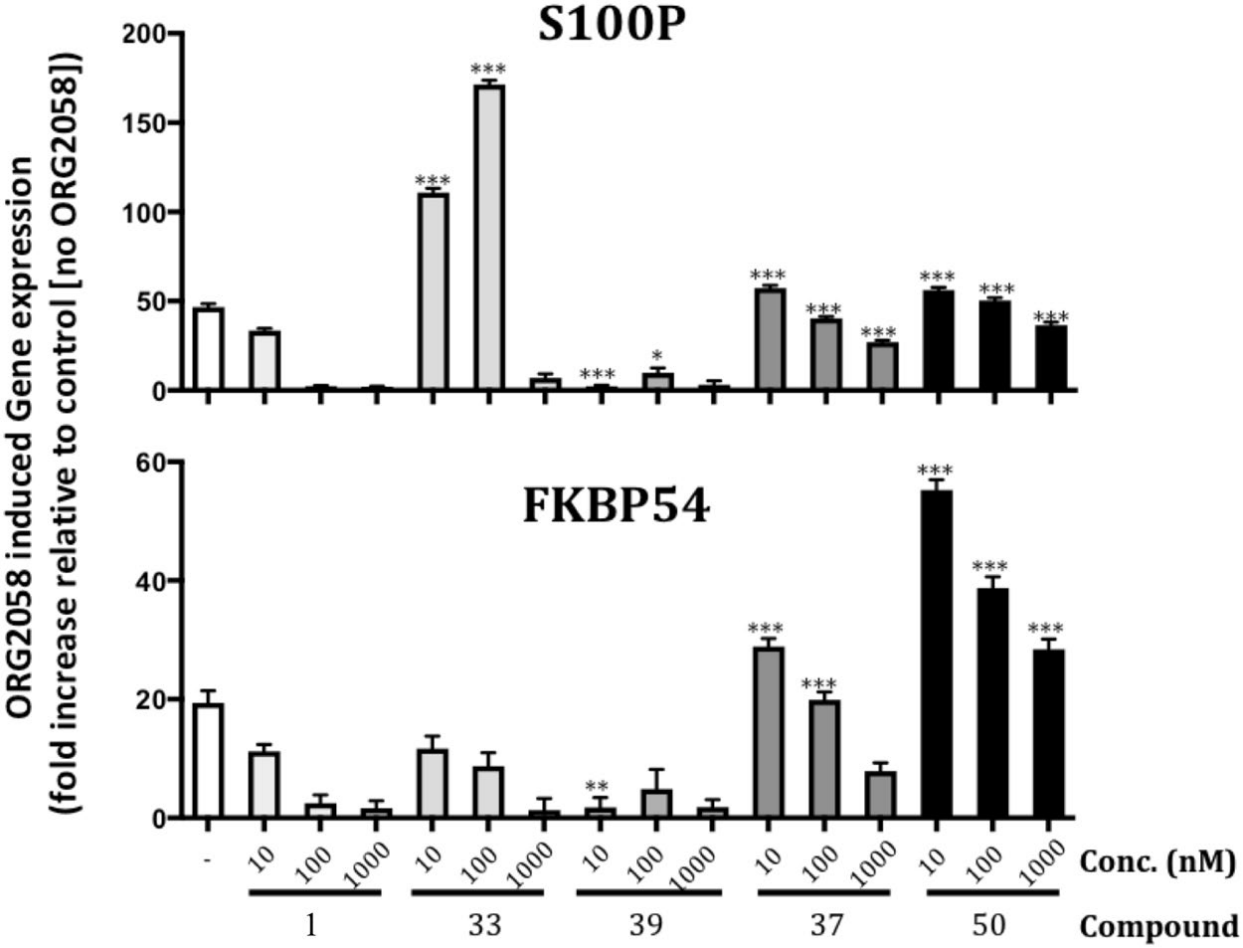
Significantly reduced anti-progestin activity in mifepristone analogues. T-47D cells were treated for 24 h with vehicle or 10 nM ORG2058, with or without increasing concentrations of compounds, followed by harvesting and RNA extraction. Transcript levels of FKBP54 and S100P, were normalised using the housekeeping gene TBP, and levels in treated samples are expressed as fold change relative to the matched vehicle-treated (ie, no ORG2058) control (normalised to 1). Data is mean ± SEM (n = 4). Asterisks denote significant difference from mifepristone treated cells, matched for concentration. *p < 0.05, **p < 0.01 and ***p < 0.001.

PR antagonism of **1** and analogues was assessed in T-47D breast cancer cells by measuring their effect in the presence of ORG2058, a highly active synthetic PR agonist that induces well-documented transcriptional targets including the FKBP54 and S100P genes^30–33^. To ascertain if our analogues had altered PR antagonism activity compared to that of parent compound **1**, cells were induced with 10 nM ORG2058 in the presence of increasing concentrations of **1** or analogue, and transcriptional changes in FKBP54 and S100P quantified by qRT-PCR (Fig. 3); gene expression was normalised to housekeeping gene TBP^34,35^, and relativized to the control (no ORG2058).

**1** strongly inhibited ORG2058-induced up-regulation of S100P and FKBP54 at 100 nM (Fig. 3). **39** showed significantly greater antagonism of the PR than **1** at low concentrations (p < 0.01 at 10 nM), with maximal antagonism of S100P and FKBP54 evident at 10 nM. Interestingly, **37**, the dioxolane analogue of **39**, showed significantly reduced (p < 0.001 at 10 and 100 nM) PR antagonism compared to **1**, with inhibition of S100P and FKBP54 only occurring at 1 µM, while at lower concentrations, **37** acted as a mild agonist, increasing expression of S100P and FKBP54. In similar fashion to **37**, **33** had reduced PR antagonism, requiring 1 uM for activity, while possessing agonist activity at lower concentrations. **50**, lacking the N,N-dimethylaniline moiety, lacked the antagonistic activity of **1** (p < 0.001 at all concentrations), and, like **33** and **37**, showed mild agonist activity at lower concentrations.

The results above are consistent with previous findings in that modifications to **1** can produce agonists, antagonists, or partial agonists^36–38^. This complexity of effects stems from the fact that PR ligand effects on PR-induced gene regulation do not relate only to blocking progesterone binding, but also to changes to PR conformation, specific effects on the PR isoforms, and altered interactions of the PR complex with DNA or downstream regulators (enhancers/inhibitors)^32,36–38^.

### Mifepristone alters CP localization in infected cells

**Figure 4:**
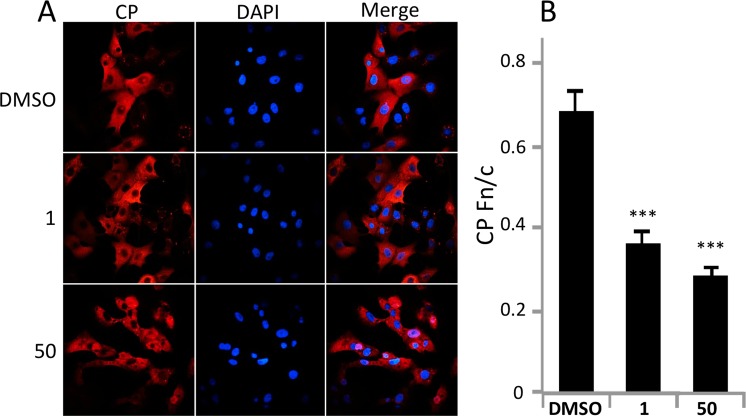
Mifepristone and analogue 50 alter CP nuclear localization in infected cells. Vero cells were pretreated with DMSO (0.1%) with or without 10 μM compound as indicated for 2 h prior to infection with VEEV-TC83 at an MOI of 0.1 in the continued presence of the respective inhibitors. At 16 h p.i., cells were fixed and probed for CP (red) and DAPI stained (blue). Images such as in A were used to quantify the ratio of nuclear to cytoplasmic fluorescence ratio (Fn/c) for CP staining in B. Data represent the mean ± SEM (n ≥ 50). ***p < 0.0001.

Compound **50** was found to inhibit the nuclear import of CP the most of the compounds tested in the FRAP assay, possessing strong anti-VEEV activity and no detectable PR antagonism activity, making it our favored compound. Its effect on CP localisation during viral infection was therefore assessed in Vero cells infected with VEEV TC-83. Cells were treated with 10 µM **1** or **50** 2 h prior to infection, followed by 16 h of post treatment. Cells were then fixed and stained for VEEV CP (Fig. 4). Consistent with the FRAP assay, both **1** and **50** reduced the nuclear accumulation of CP, confirming their activity under physiologically relevant conditions.

### Modeling progesterone receptor binding

Selective PR modulators (SPRMs) able to act as agonists or antagonists in a context dependent manner represent valuable treatment options for various hormone-dependent conditions, since they may elicit limited or negligible undesirable off-target effects compared to full antagonists and/or agonists^39–41^.

**Figure 5:**
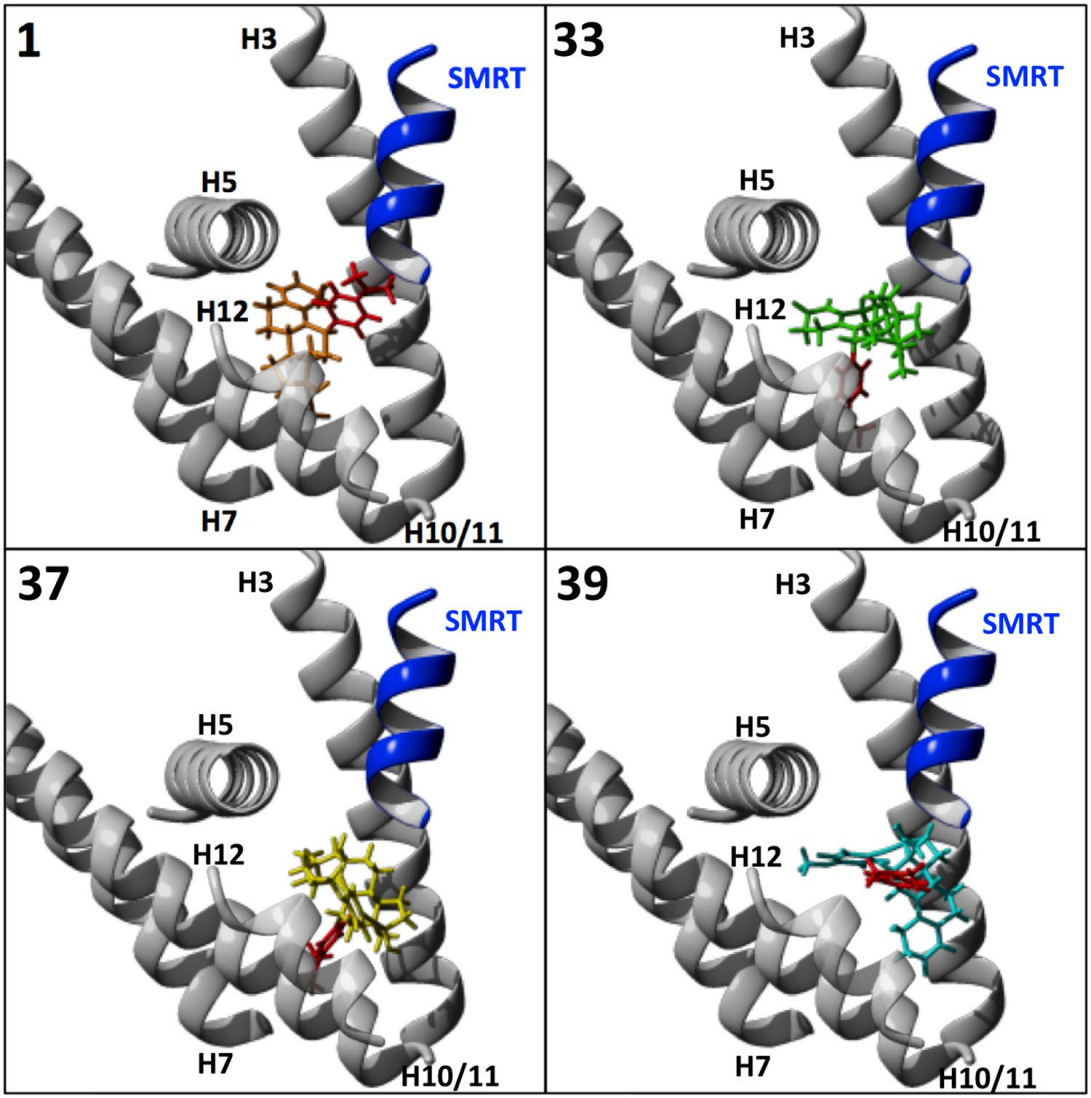
Molecular modelling of docking of mifepristone analogues to the progesterone receptor ligand binding domain in complex with corepressor Silencing Mediator of Retinoic Acid and Thyroid Hormone Receptor (SMRT). Compound **1** and analogues **33**, **37**, and **39** were docked into the LBD of the PR (grey) complexed with the SMRT peptide (blue) using Glide Docking from the Maestro suite (Schrodinger) and PDB 4OAR. Helixes (H) 3, 5, 7, 10/11, and 12 of PR are shown, with the 11-position for each compound coloured red.

**Figure 6:**
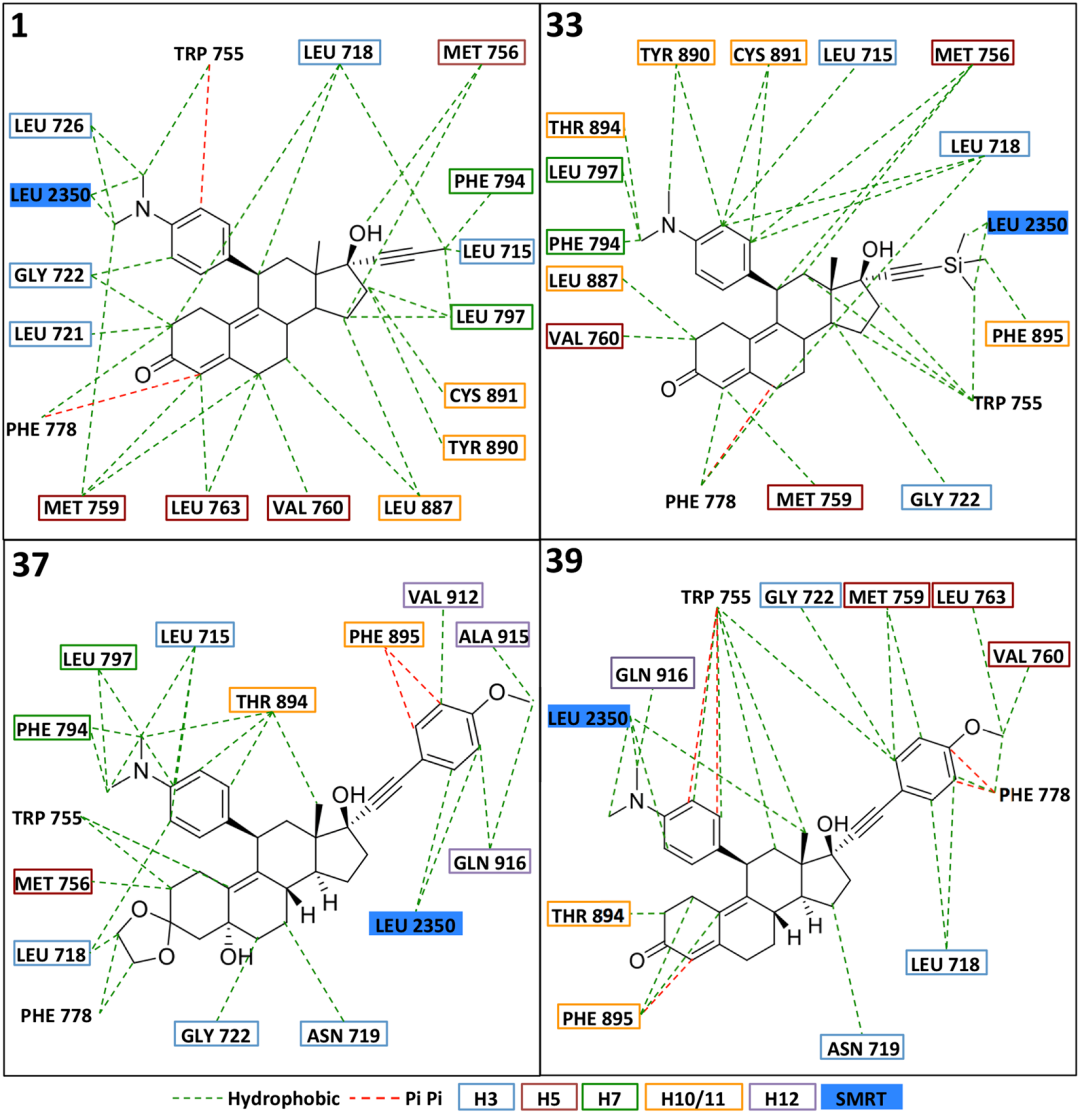
Summary of results for molecular docking of mifepristone analogues to the ligand binding domain of the progesterone receptor in complex with co-repressor SMRT. Hydrophobic and Pi Pi interactions (green and red dotted lines, respectively) between analogues and the PR/SMRT complex predicted from docking as per Fig. 5 (see Material and Methods) are depicted. Coloured boxes indicate the position of the residue (blue, H3; red, H5; green, H7; yellow, H10/11; purple, H12; and filled blue indicates the SMRT co-repressor).

To explore factors involved in the range of effects seen on the PR which may contribute to the design of SPRMs in the future, binding of **1** and the 4 analogues in the PR ligand binding domain (LBD) were modeled *in silico* using Maestro (Schrodinger, New York City, New York, USA, see Materials and Methods). Docking was performed using the crystal structure of ulipristal acetate bound to the PR LBD complexed with a peptide from the silencing mediator for retinoid and thyroid receptor (SMRT) transcriptional co-repressor (PDB ID: 4OAR)^42^; **1** docked in the LBD pocket comprising helices (H) 3, 5, 7, 10/11, 12, and the SMRT peptide, with **33**, **37**, and **39** all also able to be docked successfully in this pocket (Fig. 5). **50**, however, was unable to be docked into the PR:SMRT complex, suggesting its lack of PR antagonist activity is due to inability to bind to the PR LBD in the antagonistic formation because of the lack of the 11-position dimethylaminophenyl moiety. For the analogues that docked, there was a clear difference in the orientation in the binding pocket of **1** and **39** (no detectable PR agonistic activity), and **33** and **37**, which were partial agonists. **1** and **39** bound with the 11-position dimethylaminophenyl moiety associated with H3 and SMRT, while **33** and **37** were in an inversed orientation, with the 11-position residues nearer to H10/11 (Fig. 5). The analogues all interacted with H3, H5, and H10/11, with **37** and **39** also interacting with H12. **1**, **33**, and **37** also associated with H7; **39** did not appear to interact with H7, suggesting that this may modulate the antagonistic activity of **1** and related compounds (Fig. 6).

**39** extended further out of the pocket than **1**, while possessing extensive interactions with Leu2350 of SMRT (Figs 5 and 6). This could help stabilize the binding of the SMRT corepressor to PR, thereby enhancing PR antagonism by **39**. The 17-positon moiety of **37** extended out of the pocket to the same extent as the 11-positon of **39**, but more interactions were formed with helix 12 than with SMRT. By stabilizing H12, **37** could inhibit the ability of the PR to adopt a fully antagonistic conformation, leading to the reduced antagonism seen. **33** was a partial agonist, with reduced antagonism resulting from fewer interactions with SMRT. Given its strong agonism at low concentrations, it is also possible that **33** interacts favorably with co-activators of the PR.

### Modeling glucocorticoid receptor binding

**Figure 7:**
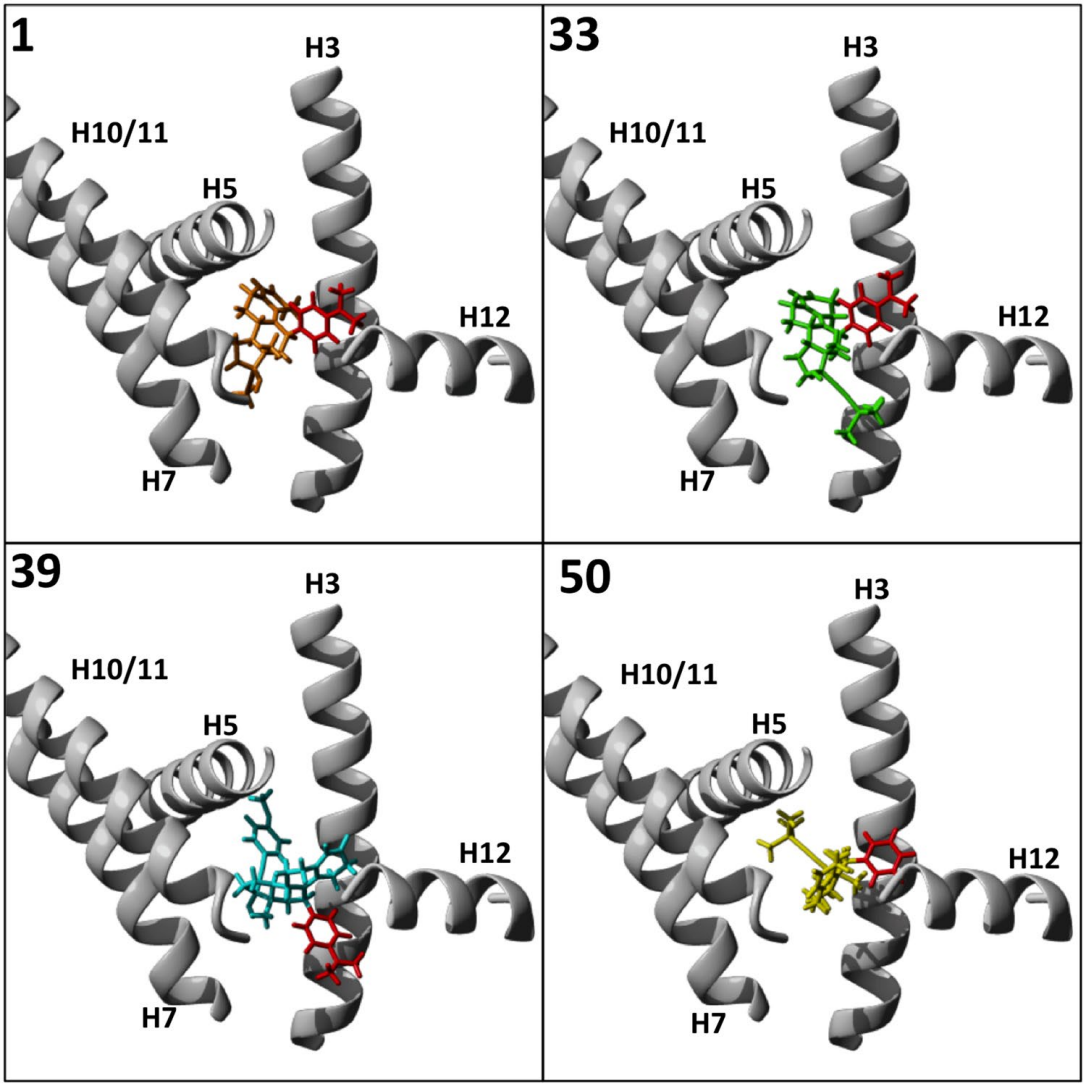
Molecular modelling of docking of mifepristone analogues to the glucocorticoid receptor ligand binding domain. Compound **1** and analogues **33**, **39**, and **50** were docked into the LBD of the GR (grey, PBD ID: 1NHZ) using Glide Docking from the Maestro suite (Schrodinger) and PDB 1NHZ. H 3, 5, 7, 10/11, and 12 are shown, with the 11-position for each compound coloured red.

As well as variable effects on the PR, the ability to target other nuclear receptors may also be desirable^43^. Glucocorticoid receptor (GR) antagonism activity of **1** is clinically important intreatment of Cushing’s syndrome^44^, for example, and provides anti-HIV activity through its ability to antagonize the GR and inhibit Vpr-induced transactivation of the HIV LTR^45^. As above, docking of **1** and its analogues to the crystal structure of the antagonist form of the GR (PDB ID:1NHZ) was performed using Maestro (see Materials and Methods)^46^. In this conformation, **1**, **33**, **39**, and **50** could be docked, but **37** could not (Fig. 7). This would suggest that **37** does not possess antagonistic activity against the GR.

Compounds **1, 39**, and **50** all docked with the 11-position domain facing towards H12 and the binding pocket opening. However, while the 17-position of **1** angled back towards the space between the bottom of H7 and H3, the 17-position of **50** extended back toward H5, where the B-ring of **1** sits. The A- and B-rings of **50** instead project out of the pocket, between H10/11 and H12. This may interfere with H12 folding into the agonist conformation, and so may increase antagonism compared to **1**. The 11-position and the A-D rings of **33** are comparable to those of **1**, but the 17-position moiety extends out of the binding pocket to the space between H3 and H12; how this may affect GR activity is unclear. While it seems unlikely that it could inhibit H12 access to the agonist position, it could conceivably stabilize H12 in either the agonist or antagonist conformation, enhancing either or both activities. The 11-position of **39** extends outward from the binding pocket to the same space between H3 and H12 as position-17 of **33**. The 17-position of **39** sits deeper in the binding pocket than any other analogue, with the methoxy group extending back below H5. Combined with the extended occupancy at the LBD opening, it is reasonable to expect **39** to possess enhanced GR antagonism compared to **1**.

## Discussion

This study describes the synthesis of a series of novel and previously reported analogues of mifepristone, and identifies multiple key structures in the pharmacophore of mifepristone. We show that the 11- and 17−positions of mifepristone are critical to its anti-VEEV activity, and that replacing the ketone moiety with a dioxolane could enhance activity depending on the context. Electrophilic hydrogen bond donors at either the 17−position or on a substituted aromatic at the 11−position resulted in a loss of antiviral activity, with more hydrophobic moieties increasing activity. Importantly, we were successful in producing a number of analogues, some of them for the first time, with significantly increased activity compared to mifepristone, with many of our compounds possessing EC_50_s in the 1–10 µM range. While the addition of bulky hydrophobic groups at either the 11- or 17-positions improved the EC_50_ of compounds, the presence of these groups at only the 11-position resulted in a strong ability to inhibit the production or infection viruses, as measured by the plaque assay.

Consistent with previous work^10,11^, compounds unable to inhibit the nuclear import of CP, and therefore its cytopathic effects, were consistently less active in plaque assays, supporting the idea that the VEEV pathogenicity is linked to CP nuclear import (see Fig. 2D). It should be noted that the mechanism of inhibition of CP nuclear localization is unlikely to be through direct impact on CP or Impα/β1 (data not shown; see)^8^ or CP-Impα/β1 binding^7^, bur is consistent with the idea that CP nuclear trafficking can be inhibited at a point other than the Impα/β1:CP interaction. Clearly, some of the compounds shown here to be unable to inhibit CP nuclear trafficking are able to limit VEEV replication in the luciferase activity, underlining the fact that there are likely other mechanisms of anti-VEEV action that can be elicited by mifepristone/mifepristone analogues.

Significantly, we succeeded in modulating mifepristone’s PR antagonistic activity by removing the N,N-dimethylaniline moiety, modifying the 17−position domain, and through masking ketones as dioxolanes. Most importantly, we have succeeded in generating mifepristone derivatives that have increased anti-VEEV activity and reduced PR antagonistic activity, demonstrating that the anti-VEEV activity of **1** and its analogues is independent of PR antagonism. Docking experiments showed that the removal of the N,N-dimethylaniline moiety prevented binding of **50** to the PR in an antagonistic formation, while other structural modifications were found to alter the orientation of analogues in the LBD, greatly reducing the strength of antagonistic activity.

Modeling of compound docking to the GR LBD showed that the mifepristone analogues examined were either unable to dock in an antagonistic structure, or docked in an altered orientation. Further studies to assess how these changes may alter GR activation and anti-HIV activity, and their potential use as SPRMs or selective GR modulators would be of great interest for other applications.

**Figure 8:**
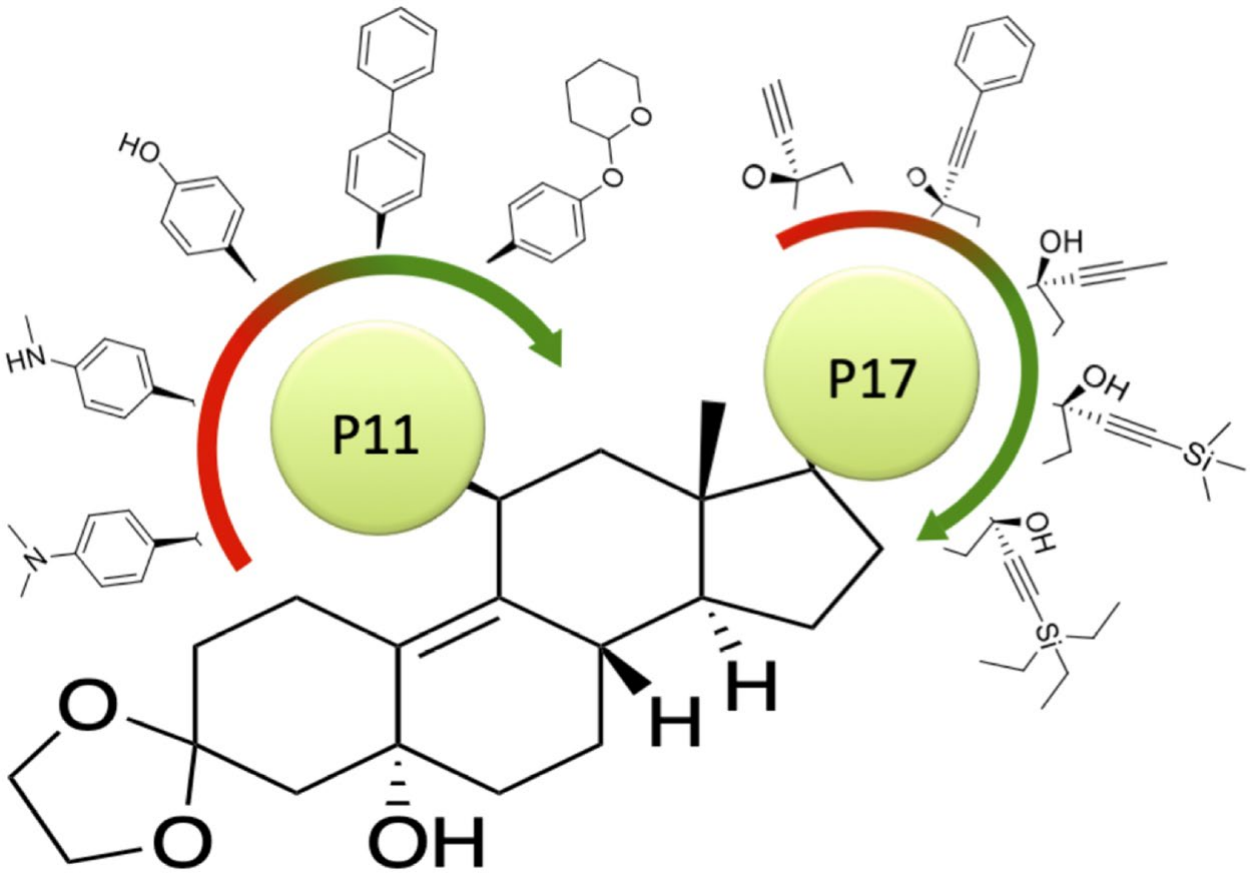
Suggested avenues for future development of mifepristone analogues. Based on the results here, further development of mifepristone analogues for anti-VEEV activity should involve retention of a dioxolane backbone, terminal aromatic ring at the 11-position, and a bulky branched moiety at the 17-position. Representative moieties are shown in order from most to least preferable (green to red).

Based on the improvements in activity, general low toxicity, and reduction in off-target effects, the compounds described here, and **50** in particular, represent an important step toward effective anti-VEEV agents that lack PR antagonism. Our SAR has identified important moieties and combinations thereof that favor anti-VEEV activity, and so present a valuable guide for future work. Further developments from the mifepristone core should possess a dioxolane backbone and terminal aromatic structure at the 11-position. They should also lack a dimethylamino moiety at the 11-position and contain bulky hydrophobic, but not aromatic, domains at the 17-position (Fig. 8). These combinations are most likely to lead to compounds with strong inhibition of VEEV replication and infectious virus production, while also reducing their effects on the PR.

## Materials and Methods

### Fluorescence recovery after photobleaching (FRAP)

For transfections, pcDNA3.1_VEEVC-GFP, containing the full length VEEV CP fused to GFP was used^10,11^. HeLa cells were maintained in Eagle’s Minimum Essential Medium (EMEM) supplemented with 10% fetal calf serum (FCS), 1 x GlutaMAX (Thermofisher, USA), and 1 x NEAA (Thermofisher) at 37 °C with 5% CO_2_. Cells grown on glass coverslips were transfected with 1 µg of pcDNA3.1_VEEVC-GFP using lipofectamine 3000 (Invitrogen) as per the manufacturer’s instructions. 22 h later, medium was replaced with medium containing DMSO with or without 200 µM compound, cells incubated for 2 h at 37 °C, and then imaged by confocal laser scanning microscopy (CLSM), using an Olympus FV1000 microscope equipped with temperate control and a 100x oil immersion lens. FRAP analysis was performed as previously^26–28^, whereby nuclei were selectively bleached (12 scans at 100% laser power, 488 nm laser) and recovery monitored every 20 s for 580 s. Digitised images were analysed using ImageJ software (National Institute of Health, Bethesda, MD) to determine the % recovery of nuclear fluorescence (Fn(rec)), calculated using the formula; Fn(rec)t_x_ = [Fn(t_x_) − Fn(t_0_)]/[Fn(pre) − Fn(t_0_)] * 100, where Fn(t_x_) is the nuclear fluorescence minus the background fluorescence at x seconds post-bleach, and Fn(pre) is the nuclear fluorescence minus background fluorescence pre-bleach.

One-phase association curves were fitted using Prism to calculate the maximal fluorescence recovery,Fn(rec)_max_. Statistical analyses were performed using a student’s t-test.

### RT-qPCR

T-47D breast cancer cells were obtained from the E.G. and G. Mason Research Institute (Worchester, MA, USA) and have been verified by STR profiling. Cells were maintained in phenol red-free RPMI1640 medium (Lonza, Melbourne, Vic) supplemented with 10% fetal calf serum (Sigma, Castle Hill, NSW) and insulin (0.25 U/ml, Novo Nordisk Australia). Cells were seeded in 25 cm^2^ flasks and allowed to attach and expand for two days, followed by treatment with progestin compounds, as indicated. After 24 h treatment, cells were harvested by trypsinization, lysed, and total RNA was extracted using Isolate II RNA isolation reagents (Bioline Australia, Alexandria, NSW). Genomic DNA contamination was eliminated by treatment of samples with RNase-free DNase I during the RNA isolation process.

RNA (0.5 μg) was reverse transcribed using Superscript II (Thermo Fisher Scientific, Melbourne, Vic) following the manufacturer’s specifications, in the presence of random hexamer (Bioline) and oligo-dT_18_ (Sigma) primers. The BioRad CFX384 Touch real time quantitative PCR platform (BioRad, Gladesville, NSW) was used to estimate expression levels of PR target transcripts in duplicate in the reverse transcribed cDNA, using Platinum SYBR Green qPCR SuperMix-UDG reagents (Thermo Fisher Scientific), and the following primers: FKBP54 – forward: 5′-TGCAGGTTTGACACGCAGTT-3′, reverse: 5′-CCTAGTCAATGGTCAGCTCAAG-3′, S100P – forward: 5′-GGTGCTGATGGAGAAGGAGC-3′, reverse: 5′-ACAGGCAGACGTGATTGCAG-3′. The TATA binding protein (TBP) transcript was used as a control for normalisation of the PCR quantitation. The primers used to amplify TBP were: forward: 5′-TGCACAGGAGCCAAGAGTGAA-3′, reverse: 5′-CACATCACAGCTCCCCACCA-3′. After normalisation of the cycle threshold values using TBP levels, transcript levels were estimated relative to the appropriate vehicle-treated control.

### CC_50_ and EC_50_ assays

CC_50_s and EC_50_s were measured as previously described by the addition of serially diluted compounds or DMSO to Vero cells and using Promega’s CellGlo Cell Viability assay or the reporter virus, VEEV TC-83 luciferase, respectively^10,11^. Compounds were added to Vero cells for 2 h prior to infection with the reporter virus, and maintained in the medium post-infection. Inhibition of viral replication was measured 16 h post-infection using Promega’s BrightGlo Luciferase assay according to the manufacturer’s specifications. Vero cells were cultured at 37 °C in a humidified 5% CO_2_ atmosphere, and all compounds described were dissolved in DMSO and diluted in DMEM (supplemented with 10% FBS, 1% L-glutamine, and 1% penicillin/streptomycin).

### Plaque Assays

Plaque assays were performed as previously^9–11,23^. Briefly, Vero cells were pretreated for 2 h before virus diluted in supplemented DMEM was added for 1 h. Virus inoculum was removed, cells were washed once with sterile 1X PBS, and media containing compound replaced. Supernatants were collected at 16 h post-infection (hpi). To determine plaque titers, crystal violet plaque assays were performed as previously^9–11,23^. Infected supernatants were serially diluted 1:10 in supplemented DMEM then placed over confluent Vero cells. A 1:1 mixture of 2× Eagle’s Minimal Essential Medium (EMEM) (supplemented with 5% FBS, 1% minimum essential amino acids, 1% sodium pyruvate, and 2% penicillin/streptomycin) and 0.6% agarose in purified water were added 1 h later to each well. After 48 h, plates were fixed with 10% formaldehyde for at least 1 h. Once the agarose plugs were removed, cells were stained with 1% crystal violet in 20% ethanol and purified water. Plaques were counted, and triplicate samples averaged.

### Immunofluorescence of infected cells

Vero cells grown on coverslips in a 6-well plate were processed for immunofluorescence analysis as previously^9^. Antibodies used were anti-VEEV-C protein (BEI Resources, NR-9403) goat primary antibody (1:1000 dilution) and Alexa Fluor 568 donkey anti-goat secondary antibody (1:500 dilution). Slides were imaged using an oil-immersion 60X objective lens on a Nikon Eclipse TE 2000-U confocal microscope. Images were then processed using the Nikon NIS-Elements AR Analysis 3.2 software. Digitized images were analyzed as previously using ImageJ 1.47 public domain software^9,10^ (see also above).

### Preparation of the 4OAR and 1NHZ structures for molecular docking experiments

Docking was carried out on the PR with bound ulipristal acetate and a peptide from the corepressor SMRT crystal structure [PDB:4OAR, 2.41 Å resolution]. To prepare the protein structure for docking, all solvent molecules were deleted and bond orders for the ligand and the protein were adjusted. The missing H-atoms were added, and side chains then energy-minimised using the OPLS-2005 force field (Maestro, Schrodinger). The ligand-binding site was defined using Receptor Grid generation (Schrodinger) centered on the bound ligand Ulipristal acetate within PDB 4OAR; default settings were used for all other parameters.

The 1NHZ structure was prepared for docking in the same manner, except that the ligand-binding site used was defined by allowing SiteMap to identify top ranked binding sites and select the one that best fit the pose of the ligand from the 1NHZ crystal structure for the Receptor Grid generation; default settings were used for all other parameters.

### Ligand preparation and docking

Mifepristone analogs were prepared using Ligprep (Schrodinger), which added H-atoms, generated ionization and tautomer states and eventually converted to low energy 3D structure with correct chiralities. For docking, all default parameters but enhance sampling (by 3) were selected. This factor allows for the generation of variations on the input structure which can be used as seed structures for conformational sampling which eventually increase the size of the docked library. Images from docking simulations were produced using YASARA (YASARA Biosciences GmbH, Vienna, Austria)^47^.

### Synthesis of mifepristone analogues

#### Epoxidation

Epoxidation of estradiene dione-3-keta (**2**) initially involved addition of H_2_O_2_ and (CF_3_)_2_CO in CH_2_Cl_2_ and stirring for 1 h, which has previously been shown to facilitate epoxidation of simple unactivated alkenes^48,49^. Methyl(trifluoromethyl)dioxirane forms *in situ*, with **2** subsequently added, and the reaction allowed to proceed at low temperature overnight to ensure complete consumption of estradiene dione-3-keta. Epoxidation was found to be completely regioselective but not stereoselective, yielding a mixture of isomers of **3** consisting of the 5*β*, 10*β*- and 5*α*, 10*α*-epoxide (35 and 65%, respectively), with the latter favoured by low temperature. Separation of the epoxides was not critical, since the final step of the synthesis resulted in elimination of the 5*β*-hydroxy formed after Grignard attack of the resultant epoxide. However, chromatographic separation of the corresponding epoxides was still carried out using flash chromatography and the 5*α*, 10*α*-epoxide was carried forward to the Grignard addition.

#### Grignard Addition at the 11-position

The Grignard reagent 4-(N,N-dimethylaniline)magnesium bromide was freshly prepared *in situ*, and subsequently added to a solution of the epoxide **3** and CuI at low temperature. In contrast; adding the epoxide and CuI directly into the Grignard solution resulted in reduced yields and increased by-products which proved difficult to resolve by ^1^H-NMR spectroscopy. The reaction of Cu-catalyzed Grignard reagent with steroidal epoxides of type 1 proceeded with complete regio- and stereospecificity, leading exclusively to 11 *β*-substituted compounds^49–52^. After reaction completion the desired compound, **4** was isolated via flash chromatography.

#### Lithiation at the 17-position

We were unable to replicate previous reports using Grignard addition at the 17-ketone, and instead used a lithiation reaction of the terminal alkyne and subsequent nucleophilic attack of the 17-position carbonyl group to alter the 17-position domain. Derivatives synthesized by this method subsequently facilitated the synthesis of other derivatives, or were further functionalized through Sonagashira coupling.

#### Diketal hydrolysis and 5*β*-hydroxy elimination

The final step in the synthetic strategy of deketalisation and 5*β*-hydroxy elimination was achieved through acid catalyzed elimination and deprotection.

Mifepristone and known steroid derivatives were synthesized as examples of analogues with different substituents at the 17-position whilst maintaining the 4-N,N-dimethylaniline moiety at the 11-position. These analogues included Ulipristal Acetate (**21**)^53,54^, Desmethyl-mifepristone (**6)**^55^, Trifluoromethylmifepristone (**9**) (where synthesis was modified from that described for a similarly related compound)^56^, along with Lilopristone (**13**) and Aglepristone (**7**); although they are commercially available, synthesis of the latter compounds has not been described, making the present study the first to report a method for their synthesis.

The synthesis of mifepristone has been well documented within the literature^57^ (Supp. Fig. S1). 1-propyne was bubbled gently through a solution of anhydrous THF at −78 °C, and *n*BuLi added drop-wise upon saturation. The reaction was then stirred and **4** was added; the reaction then quenched and carried onto the next step without further purification. The resulting diketal compound **5** was subsequently hydrolysed using aqueous acetic acid to give **1** in a 7.3% yield over two steps.

**1** was also demethylated to generate the desmethyl analogue **6** in an 8.4% yield using CaO and I_2_ in a mixture of MeOH and THF^47^ (Supp. Fig. S1). Unfortunately, this reaction gave poor conversion, yielding numerous by-products which were difficult to isolate from the desired compound. Various procedures from the literature were adapted in an attempt to improve the yield and reduce difficulties in the isolation of the desired desmethylmifepristone (**6**), including TPAP, NMO^58^, Pb(OAc)_4_, HOBt^59^ and TiCl_4_ ^60^ without success, with most procedures returning unreacted starting material.

The conversion of mifepristone to Aglepristone (**7**) was conducted under 1 atm of H_2_ using Rosenmund’s catalyst (Pd/BaSO_4_) (Supp. Fig. S1). The isolation gave a number of undesired by-products, which could not be resolved using flash chromatography. However, we were able to isolate the desired cis-alkene **7** in a 19% yield.

We also explored the addition of a trifluoromethyl group to mifepristone, as these are commonly described within literature as a suitable bio-isostere of methyl groups which often result in improved activity due to the trifluoro inductive effect^61–63^. Synthesis of the trifluoromethylpropynyl analogue **9** from **4** was conducted using LDA and 2-bromo-3,3,3-trifluoropropene by elimination of the vinylic bromide, terminal deprotonation and subsequent attack of the 17-position ketone to give the trifluoromethyl acetylide (Supp. Fig. S1)^56^. The diketal intermediate **8** was achieved with a 21% yield, 52% remaining as starting material; this was subsequently stirred in 70% AcOH to give **9** with a 66% yield, corresponding to an overall yield of 14%.

Lilopristone (**13)** was also synthesized from **4** (Supp. Fig. S1). Lithiation of THP-protected propargyl alcohol followed by reaction with **4** produced the intermediate **10**, which was carried forward without further purification. **10** was reduced using Rosenmund’s catalyst to give **12**, which was subsequently hydrolyzed to deliver Lilopristone with a 67.5% yield over two steps (hydrogenation and deprotection). Compound **10** was also stirred in 70% AcOH to give the propargyl alcohol derivative **11** with a 25% yield.

We found in most cases, the synthesis of each of these analogues often resulted in either unreacted starting material or numerous by-products which could not be isolated individually by flash chromatography. Despite the low overall yields of the desired steroid derivatives, there was sufficient material for biological evaluation (see below).

Supplementary Fig. S2 describes the most recent literature precedence for the synthesis of Ulipristal Acetate (**21**), beginning with the cyanohydrin diene-3-one^53^. Unfortunately, whilst the steps according to literature worked sequentially to deliver **17** in a good yield, TMS deprotection of **17** with HCl in acetone at 50 °C, resulted in two products **19** and **20** in the ratio of (5:3), respectively.

Synthesis of Ulipristal Acetate has been described by a number of research groups with varying methodology^53,54,64–66^, with successful synthesis of 21 achieved by adapting the approach from Dancsi *et al*.^64^, Kim *et al*.^65^, and Yu *et al*.^54^ (Supp. Fig. S3). Compound 4 was added to a solution of TMSacetylene and *n*BuLi in THF and following the addition of the TMSacetylide functional group at the 17-position, the crude material was stirred in a solution of TBAF in THF to remove the silyl protecting group and yield the terminal alkyne 22. This was subsequently reacted with 4-nitrophenylsulfenyl chloride to generate the allene 23 with a 44.9% yield, which was stable under flash chromatographic conditions. 23 was then reacted with sodium methoxide (25% in methanol) at 70 °C, followed by addition of trimethylphosphite and stirring for an additional 2 h. We were ultimately able to isolate 24 after flash chromatography, which was followed by the hydrolysis of 24 with HCl in methanol at room temperature. Hydrolysis/hydroxyl elimination of 24 did not go to completion, meaning that the crude material was carried to the next step, with hydrolysis successfully achieved using AcOH at 50 °C, to deliver 25 with a 76.9% yield. Acetylation of 25 under the conditions described by Wu *et al*., using perchloric acid and acetic anhydride in CH_2_Cl_2_ only returned starting material^54^. Instead, 25 was added to AcOH and *p*TsOH in CH_2_Cl_2_ and the reaction allowed to proceed for 30 min, before purification by flash chromatography to give 21 with a yield of 24.7%.

### Synthesis of 17-Cyano and 17-hydroxy-17-trifluoromethyl analogues

The range of tolerated moieties at the 17 position was next tested though the synthesis of 17*α*- and 17*β*- cyano analogues (**26** and **27**) and 17-hydroxy-17-trifluoromethyl analogues (**28** and **29**) from **4** (Supp. Fig. S4). Synthesis of **26** and **27** was adapted from the procedure by Cook *et al*.^67,68^. Conversion of the 17-ketone by reductive nitrilation^69^ was achieved with TosMIC (the Van Leusen reaction) to give the intermediate 17-cyano epimers with a 84% yield and in a 1.15:1 ratio; these were isolated individually and subjected to acid hydrolysis and elimination with AcOH. Compounds **26** and **27** were synthesized in 33 and 25% yield respectively with epimer **26** characterized as the 17*α* trans epimer in relation to the 13*β-*methyl and **27** characterized as the 17*β* cis epimer. This was concluded by virtue of the 13*β*-methyl shift of the 17*α* epimer having an upfield ^1^H signal at δ 0.532 compared with the 17*β* epimer, which has a ^1^H signal at δ 0.632. This is consistent with the mechanistic explanation that protonation of the intermediate 17-cyano anion generated in the TosMIC reaction would preferentially occur from the *α-*side and is in accordance with the findings that steroidal angular methyl groups undergo greater downfield shifts upon introduction of a vicinal *cis-* than of a *trans-* cyano group^68,70^.

**28** and **29** were synthesized using Ruppert’s reagent (trifluoromethyltrimethylsilane) in the presence of a fluoride ion source (Me_4_NF) in THF. Ruppert’s reagent is a good source of the trifluoromethyl anion, which is able to attack the ketone of **4** and deliver **28** and **29**. This method was adapted from the work of Cleve *et al*., and Wang *et al*.^71,72^. The intermediate diketal compound, however, was not stable, and instead of the silyl ether described by Wang *et al*., we found the 17-position to be occupied by a hydroxyl and trifluoromethyl moiety. The reaction gave an epimeric mixture of compounds in ~1:1 ratio, which were isolated individually by flash chromatography in an overall yield of 44%. Storage of the diketal intermediate led to degradation through elimination of the 5*β*-hydroxy group, but we were able to generate **28** and **29**, with a 18% overall yield across both steps. Although **28** and **29** were isolated separately we could not define the stereochemistry at the 17-position.

### Synthesis of 17- TMS, TES and TIPS acetylide analogues

We focused on introducing hydrophobicity at the 17-position since the analogues generated above with hydrophilic groups had reduced activity whilst those with hydrophobic groups retained activity. We synthesised the 17-position silyl derivatives **30**, **31**, **32** (TMS, TES, and TIPS dioxolane analogues, respectively), and **33**, **34** and **35** (TMS, TES, TIPS ketone analogues, respectively) from **4** (Supp. Fig. S5). The yields for the synthesis of intermediate silyl derivatives **30**–**32** were modest, with the reaction often returning product and starting material, which was recycled. The intermediates were subsequently acidified in AcOH at 50 °C, resulting in deketalisation and elimination of the 5*β*-hydroxy group to give **33**–**35**. The silyl group of TMSacetylide intermediate **30** was removed by treatment with TBAF in THF to give the terminal alkyne **22**, which was functionalized by Sonagashira reaction to give **36** and **37** (Supp. Fig. S5), which returned product in moderate yield along with unreacted starting material, which was recycled. Importantly Sonagashira coupling on the 11*β*-phenyl analogue of **4** gave the desired compound in good yield, but the compound degraded upon storage, even following deketalisation. The 11*β-*dimethylaminophenyl moiety appears to be essential to maintain stability for 17-position phenylacetylide analogues. Compounds **36** and **37** were subsequently stirred in AcOH to give the final compounds **38** and **39** with 96.2 and 72.7% yields respectively.

### Synthesis of 11*β-*aryl analogues

Lastly, we synthesized a series of analogues possessing a 17*β-*trimethylsilylacetylide moiety, and varied substituted and un-substituted aryl moieties at 11*β-*position (Supp. Fig. S6). Aryl moieties were installed at the 11-position through copper catalyzed Grignard addition of **3**. Grignard reagents were prepared *in situ* with the substituted bromobenzene and subsequent copper(I) catalyzed attack of the 11-position to give the 11*β-*aryl moiety. We were able to synthesise the phenyl, *para-*anisole, *para-*phenol as the THP ether, biphenyl and the *meta-*N,N-dimethylaniline analogues (**40**–**44**). Compounds **40**–**42** were isolated by flash chromatography in contrast to the biphenyl and aniline compounds (**43** and **44**) which were unstable. The yields of the intermediates **40**–**44** was 29–67% with the remainder of the isolated products encompassing unreacted starting material, which was recycled. Intermediates **40**–**44** were then functionalized at the 17-position through the lithiated TMSacetylide. In most cases, the reaction proceeded well to give the desired intermediates **45**–**49**. The *meta*-aniline gave the poorest yield of the desired 17*β*-trimethylsilylacetylide analogues, which we attribute to the possible steric effect of the meta-dimethylamino group hindering the attack of the lithiated TMSacetylide resulting in a poor yield of **49**. Lastly intermediates **45**–**49** were hydrolysed to give the desired compounds **50**–**54**. The purity of all final compounds used for testing was confirmed by LCMS analysis and determined to be ≥ 95%. More detailed discussion of the compounds can be found in Supplementary Methods.

## Supplementary information

Supplementary info

## Data Availability

Data is available upon reasonable request.
