# Supplementary material for: Novel RU486 (mifepristone) analogues with increased activity against Venezuelan Equine Encephalitis Virus but reduced progesterone receptor antagonistic activity
Source: Sci Rep. 2019 Feb 22;9:2634. doi: 10.1038/s41598-019-38671-y (PMC6385310; doi:10.1038/s41598-019-38671-y)
Supplement: Supplementary file 1 — Supplementary info [file 41598_2019_38671_MOESM1_ESM.pdf]

Novel RU486 (mifepristone) analogues with increased activity against Venezuelan Equine Encephalitis Virus but reduced progesterone receptor antagonistic activity

Aaron DeBono[#1], David R. Thomas[+1], Lindsay Lundberg[o], Chelsea Pinkham[o], Ying Cao[❖], J. Dinny Graham[❖], Christine L. Clarke[❖], Kylie M. Wagstaff[+], Sharon Shechter[x], Kylene Kehn-Hall[o], and David A. Jans[*+]

[x]Shechter Computational Solutions, Andover, MA, USA

[+]Nuclear Signaling Laboratory, Department of Biochemistry and Molecular Biology School of Biomedical Sciences, Monash University, Melbourne, Australia

[o]National Center for Biodefense and Infectious Diseases, School of Systems Biology, George Mason University, Manassas, VA, USA

[#]Medicinal Chemistry, Monash Institute of Pharmaceutical Sciences, Monash University, Parkville, Victoria 3052, Australia

[❖]Centre for Cancer Research, The Westmead Institute for Medical Research, Westmead, NSW, Australia

[1]These authors contributed equally to this work.

[*]Correspondence should be addressed to:

Prof. Jans, Nuclear Signaling Laboratory, Department of Biochemistry and Molecular Biology School of Biomedical Sciences, Monash University, Melbourne, Australia; David.Jans@monash.edu

Supplementary figures

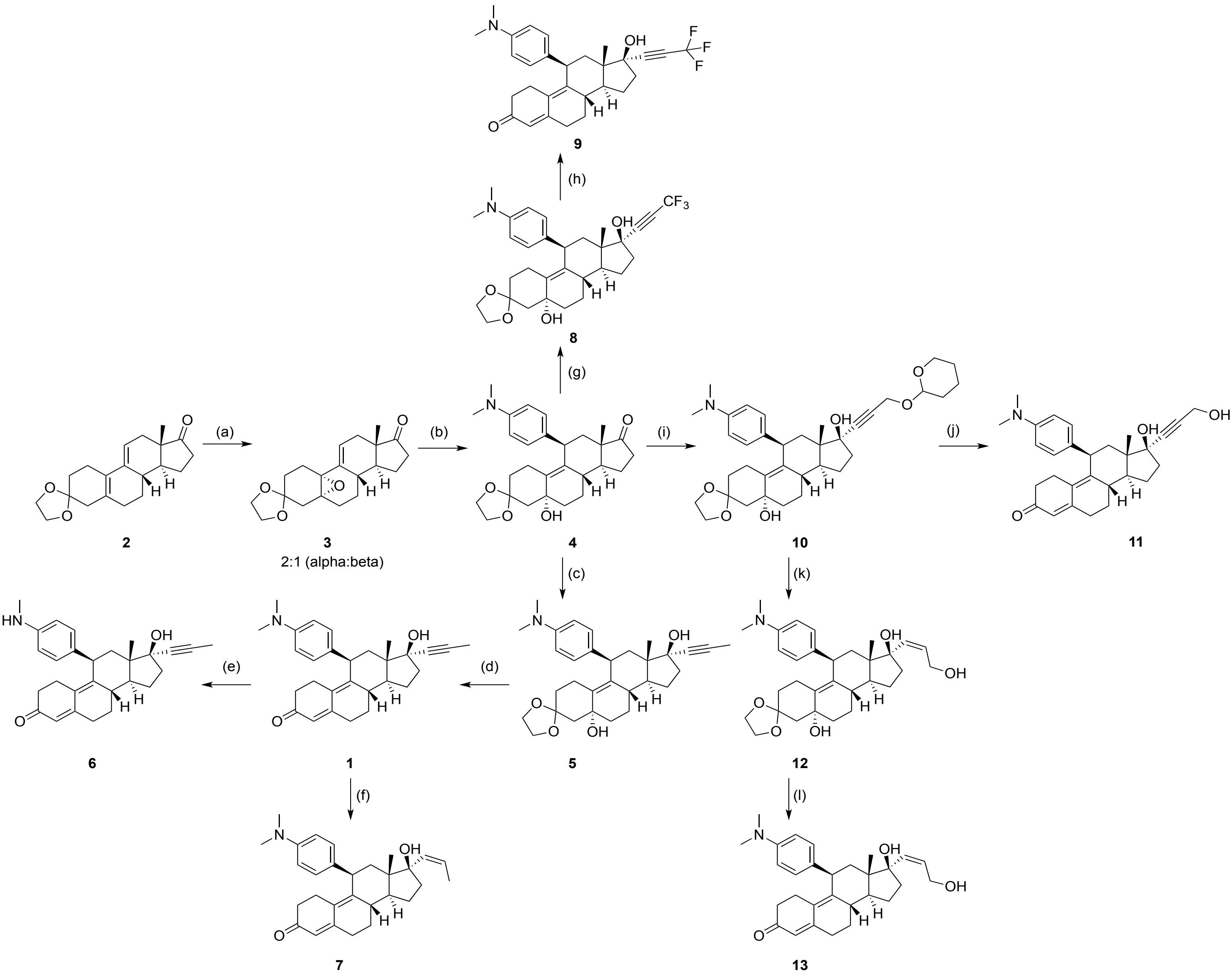

Sup figure S1: Synthesis of mifepristone (RU486) and mifepristone analogues.
Reagents: (a) H_2O_2, $(CF_3)_2CO$, Na_2HPO_4, CH_2Cl_2, 0°C, 100%, (b) $Mg_{(s)}$, $I_{2(s)}$, 4-bromo-N,N-dimethylaniline, CuI, THF, rt, 57% (c) Propyne, *n*BuLi, THF, −78°C (d) 70% AcOH, 50°C, 7.3% over two steps 1 (e) $I_{2(s)}$, CaO, MeOH: THF (1: 1), 0°C, 8.4% (f) Rosenmund's catalyst, H_2, EtOH, rt, 19% (g) LDA, 2-bromo-3,3,3-trifluro-1-propene, THF, −78°C, 21% (h) 70% AcOH, 50 °C, 66% (i) 2-(2-propynyloxy)THP, *n*BuLi, THF, −78°C, 99% (j) 70%, AcOH, 50°C, 25% (k) Rosenmund's catalyst, H_2, PhMe: EtOH (1: 1), rt (l) 70%, AcOH, 50°C, 65% over two steps.

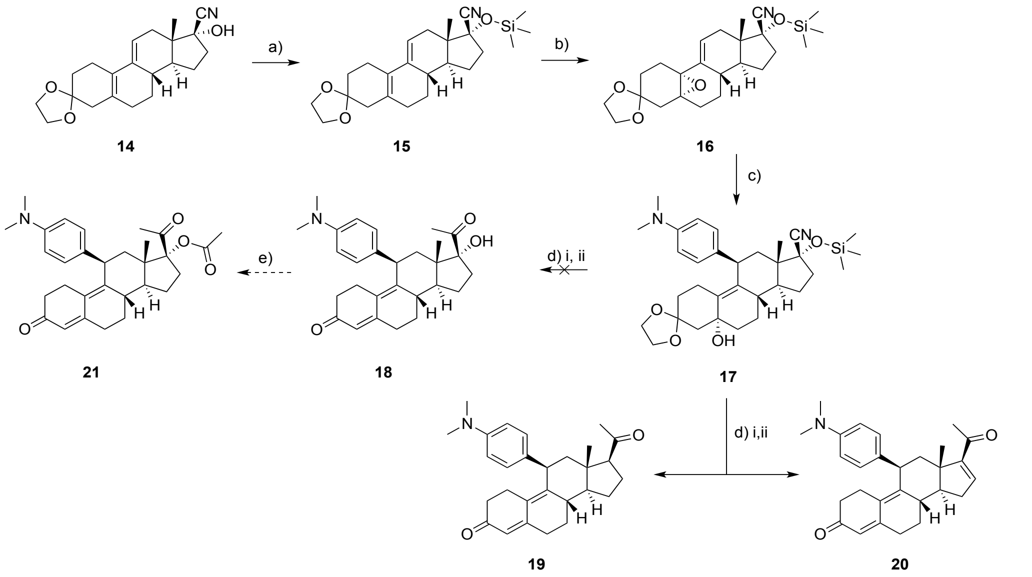

Sup figure S2: **Failed synthesis of Ulipristal Acetate**.
Reagents: (a) TMSCl, imidazole, THF, 0°C, 92.7% (b) $(CF_3)_2CO$, H_2O_2, Na_2HPO_4, CH_2Cl_2, 0°C, 67.0% (c) $Mg_{(s)}$, I_2, 4-bromo-N,N-dimethylaniline, THF, 50.3% (d) i) $Mg_{(s)}$, I_2, MeI, THF ii) HCl, acetone, 50°C, 3 h, which gave by-products (**19**: **20**) in (5:3) ratio by ^{1}H-NMR analysis and was not isolated further (e) 70% AcOH, 50°C (f) Ac_2O, $HCl0_4$, CH_2Cl_2, −30°C.

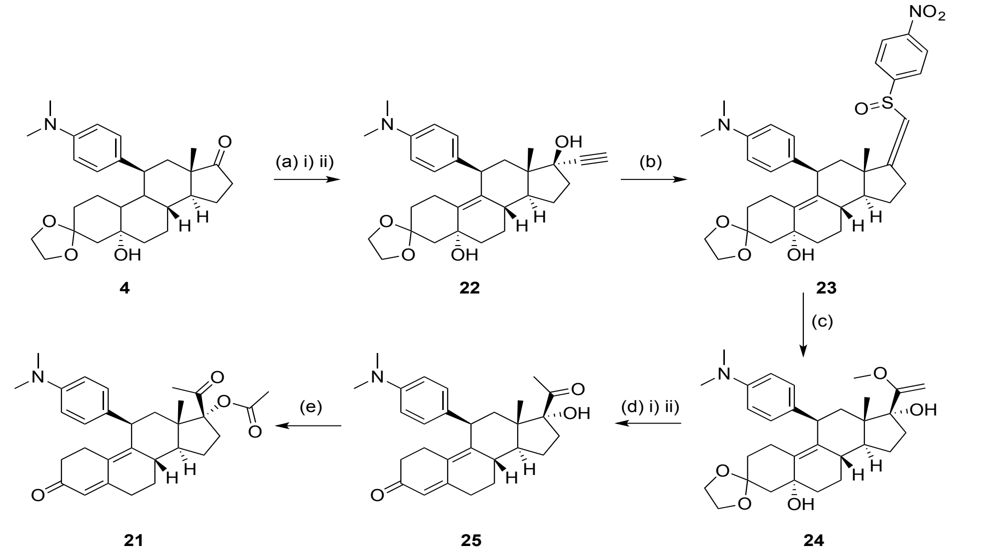

Sup figure S3: Modified synthesis of Ulipristal Acetate (21). Reagents: (a) i) TMSacetylene, *n*BuLi, THF, −78°C ii) TBAF, THF, rt , 56% over two steps (b) 4-NO_2PhSCl, Et_3N, THF, -78°C, 45% (c) CH_3ONa, trimethylphosphite, MeOH, 70°C, 28% (d) i) $HCl_{(aq)}$, MeOH, rt ii) 70 % AcOH, 50°C, 77% over two steps (e) Ac_2O, $(CF_3O)_2O$, *p*TsOH, CH_2Cl_2, −78°C, 25%.

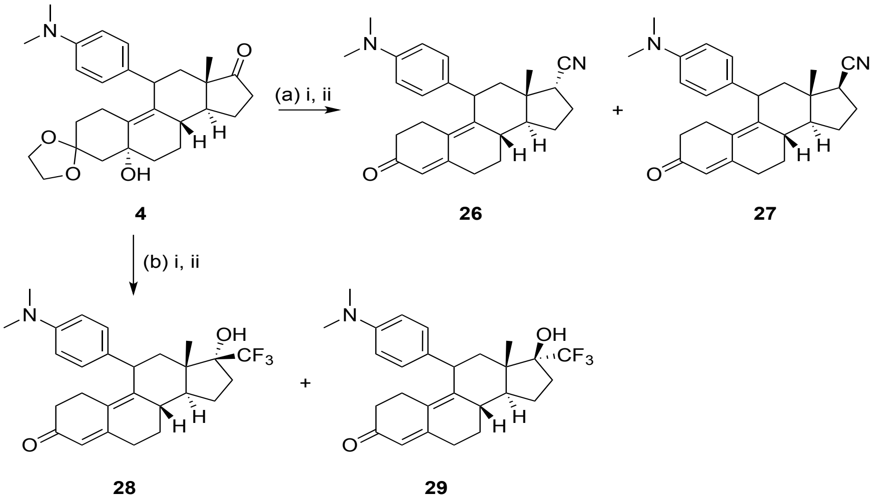

Sup Figure S4: Synthesis of 17-Cyano and 17-hydroxy-17-trifluoromethyl derivatives.
Reagents: (a) i) *t*BuOH, *t*BuOK, TosMIC, DME, 50°C ii) AcOH, 50°C, **26** (13%) **27** (12%) over two steps (b) i) trimethyl(trifluoromethyl)silane, Me_4NF, THF, 0°C, ii) 70% AcOH, 50°C (9 and 9%) over two steps.

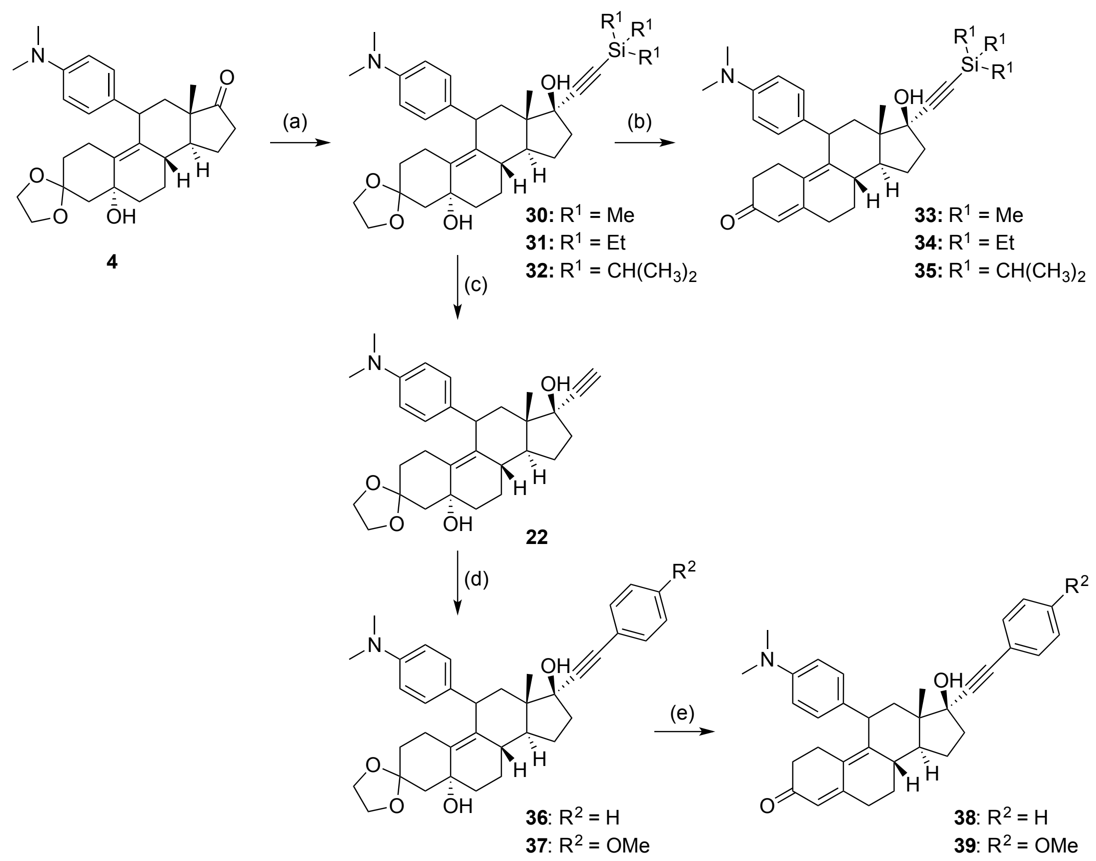

Sup fig S5: Synthesis of 17*β*-alkylsilyl acetylide analogues and novel phenyl aromatic 17*β*-side chain mifepristone analogues. Reagents: (a) **R¹** Siacetylene, *n*BuLi, −78 °C, **30** (59%), **31** (28%), **32** (17%). (b) 70% AcOH, 50°C, **33** (40%), **34** (32%), **35** (29.0%) (c) TBAF, THF, rt, 71.6% (d) **R²** ArBr, $PdCl_2(PPh_3)_2$, CuI, Et_3N, THF: H_2O, 80°C, **36** (79%), **37** (31%) (e) AcOH, 50°C, **38** (97%), **39** (72.7%).

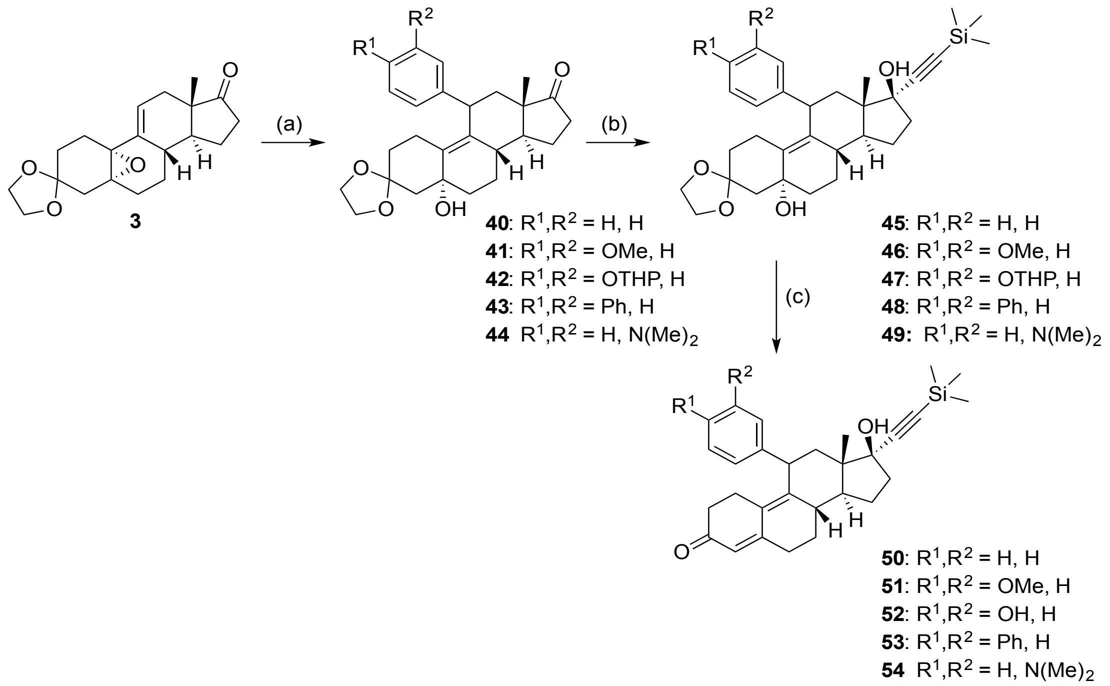

Sup fig S6: Synthesis of 11*β*-aryl, 17*β*-trimethylsilylethynyl analogues. Reagents: (a) $\mathbf{R^1}$ Bromobenzene, $Mg_{(s)}$, $I_{2(s)}$, THF, **40** (29%), **41** (47%), **42** (67%), **43**(not isolated), **44**(not isolated). (b) TMSacetylene, *n*BuLi, −78°C, THF, **45** (68%), **46** (7%), **47** (not isolated), **48** (37%), **49** (10%). (c) 70% AcOH, 50°C, H **50** (99.0%), **51**(52%), **52**(29% over two steps), **53**(60%), **54** (81%).

Supplementary methods

Small molecule NMR Spectra were recorded at 400 and 100 MHz for ^{1}H and ^{13}C respectively on a Bruker Avance 400 MHz spectrometer. NMR spectra were acquired at 298 K (each case is specified by MHz). Data acquisition and processing was managed using XWINNMR (Bruker) software package version 3.5 and plotting was managed using iNMR v5.1.1. All chemical shifts for ^{1}H NMR were measured in parts per million (ppm) referenced to an internal standard of residual proteo-solvent, δ 7.26 for chloroform. ^{13}C NMR were measured in ppm referenced to an internal standard of residual proteo-solvent, δ 77.16 for chloroform. The spin multiplicities are reported as s = singlet, br s = broad singlet, d = doublet, t = triplet, q = quartet, m = multiplet. Low Resolution Mass Spectrometry analyses were performed using a Micromass Platform II single quadrupole mass spectrometer equipped with an atmospheric pressure (ESI/APCI) ion source. Sample management was facilitated by an Agilent 1100 series HPLC system and the instrument was controlled using MassLynx v3.5. Compounds were analysed using a Waters 2690 Separation Module by RP-HPLC using a gradient of 20-100% buffer B in buffer A over 10 min followed by isocratic 100% buffer B for 6 min then gradient to 20% buffer B in buffer A over 1 min followed by isocratic 20% buffer B in buffer A for 3 min at a flow rate of 1.0 ml/min. EmpowerPro managed the running and processing of samples. Thin layer chromatography was performed on Merck Silica Gel 60 F254 plates. Thin layer chromatography (TLC) plates were visualised under UV illumination at 254 nm and/or with the aid of phosphomolybdic acid (PMA) and aniline. Column chromatography was conducted using Davisil silica gel LC60A (40-63 μm). All commercially available chemicals were purchased from Aldrich, Merck, Alfa-Aesar, Boron Molecular, and Matrix scientific and stored appropriately and used as required.

(5'*R*,10'*R*)-13'-methyl-1',2',6',7',8',12',13',14',15',16'-decahydro-4'*H*,17'*H*-spiro[[1,3]dioxolane-2,3'-[5,10]epoxycyclopenta[*a*]phenanthren]-17'-one (3)

Hydrogen peroxide (35%, 757 μl, 7.8 mmol) was added to a mixture of hexafluoroacetone sesquihydrate (1.10 ml, 7.9 mmol) in CH_2Cl_2 (20 mL) and cooled to -5°C in an ice bath. The mixture was stirred for 45 min, followed by the dropwise addition of **2** (1.5 g, 4.8 mmol) in CH_2Cl_2 (5 mL). Following the addition of diene, Na_2PO_4 (1.2 g, 8.7 mmol) was added in one portion and the reaction stirred vigorously at 0°C for 8 h. Upon completion, the reaction was diluted with CH_2Cl_2 (30 ml) and washed with 10% Na_2SO_3 (2 × 20 ml), followed by H_2O (20 ml). The organic layer was then dried over anhydrous $MgSO_4$, filtered and evaporated *in vacuo*. Flash chromatography of the crude using eluent Pet. Spirits: EtOAc (2: 1) afforded the pure 5α,10α-epoxide (1.13 g, 3.4 mmol, 75%) along with the 5β,10β-epoxide (0.4 g, 1.2 mmol, 25%). ^{1}H-NMR (400 MHz; $CDCl_3$): α-epoxide δ 6.04 (q, *J* = 3.3 Hz, 1H), 3.96-3.86 (m, 4H), 2.50-2.41 (m, 2H), 2.15-2.03 (m, 6H), 1.94-1.88 (m, 3H), 1.78-1.66 (m, 2H), 1.55-1.48 (m, 4H), 1.27-1.22 (m, 1H), 0.87 (s, 3H). ^{13}C-NMR (101 MHz; CDCl3): δ 136.7, 125.8, 107.1, 64.43, 64.23, 61.7, 60.2, 46.8, 46.4, 40.4, 37.2, 36.0, 33.7, 31.7, 28.2, 25.2, 22.3, 22.0, 14.9

(β-epoxide) ^{1}H−NMR (400 MHz; $CDCl_3$): δ 5.86 (ddd, *J* = 5.1, 3.2, 1.6 Hz, 1H), 3.96-3.87 (m, 4H), 2.51-2.44 (m, 1H), 2.32 (d, *J* = 15.3 Hz, 1H), 2.22-2.01 (m, 7H), 1.93 (dd, *J* = 15.2, 2.4 Hz, 1H), 1.85 (ddd, *J* = 14.9, 6.9, 2.0 Hz, 1H), 1.80-1.68 (m, 2H), 1.60-1.53 (m, 3H), 1.42 (ddd, *J* = 12.3, 10.6, 5.8 Hz, 1H), 1.22-1.13 (m, 1H), 0.86 (s, 3H). ^{13}C−NMR (101 MHz; $CDCl_3$): δ 138.5, 123.5, 107.2, 64.5, 64.29, 64.24, 62.4, 48.2, 46.1, 42.5, 36.3, 34.6, 33.7, 31.2, 28.1, 27.0, 24.4, 22.8, 14.4. LCMS: 331.2 $[M+H]^+$

(5*R*,8*S*,11*R*,13*S*,14*S*)-11-(4-(dimethylamino)phenyl)-5-hydroxy-13-methyl-1,4,5,6,7,8,11,12,13,14,15,16-dodecahydrospiro[cyclopenta[*a*]phenanthrene-3,2'-[1,3]dioxolan]-17(2*H*)-one (4)

Magnesium Turnings (0.16 g, 6.7 mmol) and a crystal of iodine where spot heated with a heat gun in anhydrous THF (10 mL). The temperature of the solution was maintained at ~50°C for 1

h. After activation of the magnesium surface the solution was allowed to cool to rt. 4-bromo-N,N-dimethylaniline (1.2 g, 6.1 mmol) was added dropwise in THF (10 mL) and following the complete addition the reaction was heated to 50°C for 1 h. Separately **3** (0.6 g, 1.9 mmol) dissolved in anhydrous THF (10 mL) was cooled to 0°C and $CuCl_{(s}$ (0.03 g, 0.03 mmol) was added and the reaction was stirred for 30 min. The Grignard solution was then added to this mixture dropwise at 0°C for 1 h then the reaction was allowed to heat to rt. The reaction was allowed to proceed overnight and then reduced *in vacuo*. The crude mixture was then loaded onto a silica column and flash chromatography was conducted. Eluent used Pet. Spirits: EtOAc (2: 1) delivered the title compound (0.47 g, 1.0 mmol, 57%). ^{1}H−NMR (400 MHz; $CDCl_3$): δ 7.06 (d, J = 8.3 Hz, 2H), 6.64 (d, J = 8.9 Hz, 2H), 4.37 (s, 1H), 4.24 (d, J = 6.9 Hz, 1H), 4.02-3.90 (m, 4H), 2.90 (s, 6H), 2.50-2.38 (m, 3H), 2.36-2.26 (m, 2H), 2.05-2.00 (m, 2H), 1.84-1.79 (m, 3H), 1.65-1.61 (m, 3H), 1.54-1.50 (m, 2H), 1.35-1.19 (m, 3H), 0.51 (s, 3H). ^{13}C−NMR (101 MHz; $CDCl_3$): δ 220.4, 148.6, 134.8, 134.2, 133.7, 127.7, 112.8, 108.9, 70.2, 64.8, 64.2, 50.9, 47.68, 47.54, 40.8, 38.5, 38.1, 37.76, 37.58, 35.8, 35.2, 23.54, 23.37, 22.2, 14.4. LCMS: 452.2 $[M+H]^+$

(8*S*,11*R*,13*S*,14*S*,17*S*)-11-(4-(dimethylamino)phenyl)-17-hydroxy-13-methyl-17-(prop-1-yn-1-yl)-1,2,6,7,8,11,12,13,14,15,16,17-dodecahydro-3*H*-cyclopenta[*a*]phenanthren-3-one (1)

*n*BuLi was dissolved in anhydrous THF (4 mL) within a bomb vessel and cooled to −40°C followed by the addition of 1-$propyne_{(g)}$ which was bubbled through the solution gently using a leak cylinder until saturated. Care was taken to ensure pressure did not build up through the use of an open tap. The reaction was then allowed to stir at −40°C for 15 min followed by the addition of **4** (0.05 g, 0.12 mmol) in THF (1 ml) drop wise. The reaction was then stirred at −40°C for 15 min then allowed to heat to rt and stirred for an additional 2 h. Reaction was quenched with $NH_4Cl_{(aq)}$ and extracted with EtOAc (2 ×10 ml). The organic layers was then

combined, dried over anhydrous $MgSO_4$, filtered and evaporated *in vacuo.* The crude product was then dissolved in 70% AcOH (2 ml) and heated to 50°C for 2 h. The crude reaction was then diluted by the addition of H_2O and neutralized to pH 7 by the dropwise addition of 2M NaOH. The aqueous layer was then extracted with EtOAc (2 × 10 ml). The organic layer was then dried over anhydrous $MgSO_4$, filtered and evaporated *in vacuo*. Flash chromatography of the crude using eluent EtOAc: Pet. spirits (1: 1) delivered the title compound (0.03 g, 0.07 mmol, 58%). ^{1}H−NMR (400 MHz; $CDCl_3$): δ 7.01 (d, *J* = 8.3 Hz, 2H), 6.66 (d, *J* = 8.9 Hz, 2H), 5.75 (s, 1H), 4.35 (d, *J* = 6.8 Hz, 1H), 2.91 (s, 6H), 2.80-2.74 (m, 1H), 2.59-2.56 (m, 2H), 2.49-2.40 (m, 2H), 2.38-2.19 (m, 5H), 2.04-1.93 (m, 2H), 1.89 (s, 3H), 1.80 (s, 1H), 1.76-1.67 (m, 2H), 1.51-1.31 (m, 2H), 0.54 (s, 3H). ^{13}C−NMR (101 MHz; $CDCl_3$): δ 199.7, 157.0, 148.7, 146.9, 132.2, 129.2, 127.7, 122.9, 112.9, 82.62, 82.54, 80.4, 50.0, 47.0, 40.8, 39.7, 39.3, 39.06, 39.03, 37.1, 31.3, 27.5, 26.0, 23.5, 13.8, 4.0. LCMS: 430.4 $[M+H]^+$

(8*S*,11*R*,13*S*,14*S*,17*S*)-17-hydroxy-13-methyl-11-(4-(methylamino)phenyl)-17-(prop-1-yn-1-yl)-1,2,6,7,8,11,12,13,14,15,16,17-dodecahydro-3*H*-cyclopenta[*a*]phenanthren-3-one (6)

1 (0.2 g, 0.47 mmol) was dissolved in a mixture of methanol: THF (1: 1) (3 ml) and anhydrous CaO (0.22 g, 3.96 mmol) was added. The solution was cooled to −5°C and I_2 (0.295 g, 2.33 mmol) dissolved in the above solvent was then added dropwise at 0°C for 20 min, then the reaction was allowed to stir for 1 h at 0°C after which the reaction was poured over celite and was washed with CH_2Cl_2 (20 ml). The resultant organic layer was washed with $Na_2S_2O_3$ (2 × 5 mL), then water (2 × 5 ml). The organic layer was then dried over anhydrous $MgSO_4$, filtered and evaporated *in vacuo.* Flash chromatography of the crude using eluent toluene: acetone (11: 1) delivered the title compound (0.016 g, 0.04 mmol, 8.3%). ^{1}H−NMR (400 MHz; $CDCl_3$): δ 7.00 (d, *J* = 8.1 Hz, 2H), 6.63 (d, *J* = 8.6 Hz, 2H), 5.76 (d, *J* = 0.2 Hz, 1H), 4.35 (d, *J* = 7.1 Hz, 1H), 2.83-2.80 (m, 3H), 2.80-2.73 (m, 1H), 2.59-2.56 (m, 2H), 2.49-1.94 (m, 9H), 1.89 (s, 3H),

1.78-1.69 (m, 3H), 1.51-1.32 (m, 3H), 0.54 (s, 3H). ^{13}C−NMR (101 MHz; $CDCl_3$): δ 199.8, 157.1, 147.1, 146.9, 133.2, 129.22, 129.16, 128.3, 127.8, 125.4, 122.8, 112.8, 82.63, 82.45, 80.3, 49.9, 47.0, 39.8, 39.3, 39.05, 38.99, 37.0, 31.3, 31.0, 27.5, 25.9, 23.5, 13.8, 4.0. LCMS: 416.3 $[M+H]^+$

(8S,11R,13S,14S,17R)-11-(4-(dimethylamino)phenyl)-17-hydroxy-13-methyl-17-((Z)-prop-1-en-1-yl)-1,2,6,7,8,11,12,13,14,15,16,17-dodecahydro-3H-cyclopenta[a]phenanthren-3-one (7)

1 (0.1 g, 0.23 mmol) was dissolved in EtOH (10 ml) and Pd/$BaSO_4$ was added in one portion and a hydrogen balloon was attached. The reaction vessel was devoid of oxygen through the use of a tap and positive pressure of H_2 following which the tap was closed and reaction allowed to proceed overnight under a hydrogen atmosphere. The reaction was assessed by TLC and after completion, was poured over celite to remove palladium waste. The organic layer was then evaporated in vacuo. Flash chromatography of the crude using eluent toluene: acetone (11: 1) delivered the title compound (0.019 g, 0.043 mmol, 19%). ^{1}H−NMR (400 MHz; $CDCl_3$): δ 7.04 (d, J = 8.3 Hz, 2H), 6.69 (d, J = 8.9 Hz, 2H), 5.77 (s, 1H), 5.63 (dd, J = 11.8, 7.1 Hz, 1H), 5.53 (dd, J = 11.8, 1.5 Hz, 1H), 4.33 (d, J = 7.0 Hz, 1H), 2.94 (s, 6H), 2.78-2.73 (m, 1H), 2.59-2.56 (m, 2H), 2.50-2.27 (m, 6H), 2.14-2.02 (m, 4H), 1.87-1.83 (m, 1H), 1.76-1.71 (m, 2H), 1.54-1.40 (m, 4H), 0.64 (s, 3H). ^{13}C−NMR (101 MHz; $CDCl_3$): δ 199.9, 157.2, 148.6, 147.1, 134.4, 132.3, 129.1, 127.6, 127.3, 122.8, 112.9, 85.2, 49.9, 47.8, 40.8, 39.6, 39.3, 38.9, 37.8, 37.0, 31.2, 27.5, 25.9, 23.8, 15.3, 15.0. LCMS: 432.2 [M+H]+

(5*R*,8*S*,11*R*,13*S*,14*S*,17*S*)-11-(4-(dimethylamino)phenyl)-13-methyl-17-(3,3,3-trifluoroprop-1-yn-1-yl)-1,2,6,7,8,11,12,13,14,15,16,17-dodecahydrospiro[cyclopenta[*a*]phenanthrene-3,2'-[1,3]dioxolane]-5,17(4*H*)-diol (8)

2-bromo-3,3,3-trifluoropropene was dissolved in anhydrous THF (1 ml) and cooled to −78°C, after which 2 M lithium diisoproyl amide (885 μl, 1.77 mmol) was added dropwise at −78°C and the reaction was allowed to stir for 30 min. **4** (0.1 g, 0.22 mmol) dissolved in anhydrous THF (1 ml) was then added drop wise and following complete addition the reaction was allowed to stir for 1 h at −78°C. The reaction was then allowed to warm to rt and stir for an additional 18 h. The reaction was then quenched with $NH_4Cl_{(aq)}$ and extracted with EtOAc (2 × 10 ml). The organic layers were then dried over anhydrous $MgSO_4$, filtered and evaporated *in vacuo*. Flash chromatography of the crude using eluent Pet. spirits: EtOAc (2: 1) delivered the title compound (0.025 g, 0.05 mmol, 21%). ^{1}H−NMR (400 MHz; $CDCl_3$): δ 7.04 (d, *J* = 8.4 Hz, 2H), 6.65 (d, *J* = 8.9 Hz, 2H), 4.43 (s, 1H), 4.27 (d, *J* = 6.9 Hz, 1H), 4.03-3.90 (m, 4H), 2.90 (s, 6H), 2.48-2.43 (m, 1H), 2.37-1.24 (m, 18H), 0.50 (s, 3H). ^{13}C−NMR (101 MHz; $CDCl_3$): δ 148.5, 134.5, 134.1 (*J* = 33 Hz), 127.8, 112.9, 108.9, 90.7 (*J* = 6.5 Hz), 80.1 (*J* = 1.3 Hz), 70.3, 64.8, 64.2, 50.2, 47.6, 47.4, 40.9, 39.19, 39.03, 38.65, 38.51, 38.42, 35.2, 24.2, 23.97, 23.90, 23.4, 20.9, 13.5. LCMS: 546.2 $[M+H]^+$

(8*S*,11*R*,13*S*,14*S*,17*S*)-11-(4-(dimethylamino)phenyl)-17-hydroxy-13-methyl-17-(3,3,3-trifluoroprop-1-yn-1-yl)-1,2,6,7,8,11,12,13,14,15,16,17-dodecahydro-3*H*-cyclopenta[*a*]phenanthren-3-one (9)

8 (0.017 g, 0.03 mmol) was dissolved in 70% AcOH (2 ml) and heated to 50°C for 2 h. The crude reaction was then diluted by the addition of H_2O and neutralized to pH 7 by the dropwise addition of 2M NaOH. The aqueous layer was then extracted with CH_2Cl_2 (2 × 10 ml). The organic layer was then dried over anhydrous $MgSO_4$, filtered and evaporated *in vacuo* to give the title compound without the requirement of flash chromatography (0.01 g, 0.02 mmol, 66%). ^{1}H−NMR (400 MHz; $CDCl_3$): δ 7.01 (d, *J* = 9.1 Hz, 2H), 6.66 (d, *J* = 8.9 Hz, 2H), 5.77 (s, 1H),

4.39 (d, J = 6.8 Hz, 1H), 2.92 (s, 6H), 2.82-2.75 (m, 1H), 2.61-2.57 (m, 2H), 2.52-2.45 (m, 2H), 2.39-2.32 (m, 4H), 2.23-2.17 (m, 1H), 2.05-2.00 (m, 3H), 1.86-1.77 (m, 1H), 1.51-1.41 (m, 3H), 0.58 (s, 3H). ^{13}C−NMR (101 MHz; $CDCl_3$): δ 199.8, 156.5, 148.8, 145.4, 131.6, 129.8, 127.6, 123.2, 115.9, 112.9, 90.5, 90.4, 80.0, 50.4, 47.6, 40.7, 39.5, 39.2, 39.0, 38.5, 36.9, 31.2, 27.5, 26.0, 23.6, 13.7. LCMS: 484.2 $[M+H]^+$

(8*S*,11*R*,13*S*,14*S*,17*S*)-11-(4-(dimethylamino)phenyl)-17-hydroxy-17-(3-hydroxyprop-1-yn-1-yl)-13-methyl-1,2,6,7,8,11,12,13,14,15,16,17-dodecahydro-3*H*-cyclopenta[*a*]phenanthren-3-one (11)

Tetrahydro-2-(2-propynyloxy)-2*H*-pyran (59 µl, 0.42 mmol) was added to anhydrous THF (3 ml) and cooled to −78°C. *n*BuLi (295 µl, 0.47 mmol) was added to the solution drop wise and after complete addition the reaction was stirred at −78°C for 1 h. **4** (0.05 g, 0.11 mmol) was added drop wise in anhydrous THF (1 ml) and the reaction was then allowed to warm to rt and proceed overnight for 18 h. The reaction was quenched by the drop wise addition of $NH_4Cl_{(aq)}$ and the organic layer was extracted with EtOAc (2 × 10 ml). The organic layers were then dried over anhydrous $MgSO_4$, filtered and evaporated *in vacuo*. The crude sample was subsequently carried to the next step without further purification. (0.062 g, 0.11 mmol) was dissolved in 70% AcOH (2 ml) and heated to 50°C for 2 h. The crude reaction was then diluted by the addition of H_2O and neutralized to pH 7 by the drop wise addition of 2M NaOH. The aqueous layer was then extracted with CH_2Cl_2 (2 × 10 ml). The organic layer was then dried over anhydrous $MgSO_4$, filtered and evaporated *in vacuo*. Flash chromatography of the crude using eluent Pet. spirits: EtOAc (2: 1) delivered the title compound (0.012 g, 0.027, 25%). ^{1}H−NMR (400 MHz; $CDCl_3$): δ 7.01 (d, J = 8.3 Hz, 2H), 6.66 (d, J = 8.9 Hz, 2H), 5.76 (s, 1H), 4.36 (s, 3H), 2.91 (s, 6H), 2.79-2.73 (m, 1H), 2.59-2.56 (m, 2H), 2.50-0.82 (m, 19H), 0.56 (s, 3H).^{13}C−NMR (101 MHz; $CDCl_3$): δ 199.7,156.8, 148.8, 146.4, 132.1, 129.5, 127.7, 123.0, 112.9, 89.0, 85.0, 80.3, 51.5,

50.1, 47.2, 40.8, 39.7, 39.3, 39.08, 39.02, 37.0, 31.3, 27.6, 26.0, 23.5, 13.8, 1.2 LCMS: 446.3 $[M+H]^+$

(8*S*,11*R*,13*S*,14*S*,17*R*)-11-(4-(dimethylamino)phenyl)-17-hydroxy-17-((*E*)-3-hydroxyprop-1-en-1-yl)-13-methyl-1,2,6,7,8,11,12,13,14,15,16,17-dodecahydro-3*H*-cyclopenta[*a*]phenanthren-3-one (13)

Tetrahydro-2-(2-propynyloxy)-2*H*-pyran (149 µl, 0.99 mmol) was added to anhydrous THF (6 ml) and cooled to −78°C. *n*BuLi (1.3 ml, 1.19 mmol) was added to the solution drop wise and after complete addition the reaction was stirred at −78°C for 1 h. **4** (0.12 g, 0.27 mmol) was added drop wise in anhydrous THF (1 ml) and the reaction was then allowed to warm to rt and proceed overnight for 18 h. The reaction was quenched by the drop wise addition of $NH_4Cl_{(aq)}$ and the organic layer was extracted with EtOAc (2 × 10 ml). The organic layers were then dried over anhydrous $MgSO_4$, filtered and evaporated *in vacuo*. The crude sample was subsequently carried to the next step without further purification. The crude sample was dissolved in a toluene: ethanol mixture (1: 1) (1 ml) and placed in a 3-neck flask. Pd/$BaSO_4$ was added in one portion and a hydrogen balloon was attached. The reaction vessel was devoid of oxygen through the use of a tap and positive pressure of $H_{2(g)}$ following which the tap was closed and reaction allowed to proceed overnight under a hydrogen atmosphere. The reaction was assessed by TLC and after completion was poured over celite to remove palladium waste. The crude material was carried over to the next step without further purification due to instability of intermediate **12**. Crude of **12** was dissolved in 70% AcOH (2 mL) and heated to 50°C for 2 h. The crude reaction was then diluted by the addition of H_2O and neutralized to pH 7 by the drop wise addition of 2M NaOH. The aqueous layer was then extracted with CH_2Cl_2 (2 × 10 ml). The organic layer was then dried over anhydrous $MgSO_4$, filtered and evaporated *in vacuo.* Flash chromatography of the crude using eluent toluene: acetone (5: 1) delivered the title compound (0.018 g, 0.041, 65%).

^{1}H−NMR (400 MHz; $CDCl_3$): δ 7.01 (d, *J* = 8.4 Hz, 2H), 6.66 (d, *J* = 8.8 Hz, 2H), 5.73 (dd, *J* = 12.0, 6.1 Hz, 2H), 5.64 (d, *J* = 12.4 Hz, 1H), 4.36-4.25 (m, 3H), 2.91 (d, *J* = 6.6 Hz, 6H), 2.87-2.69 (m, 3H), 2.58-2.23 (m, 6H), 2.07-2.00 (m, 3H), 1.87-1.39 (m, 5H), 0.89-0.79 (m, 1H), 0.62 (s, 3H). ^{13}C−NMR (101 MHz; $CDCl_3$): δ 199.8, 157.0, 148.7, 146.7, 136.1, 129.3, 128.7, 127.6, 122.9, 112.9, 85.7, 59.6, 50.5, 47.5, 40.8, 39.60, 39.43, 39.41, 38.5, 37.0, 31.2, 27.6, 26.0, 23.8, 15.1. LCMS: 448.3 $[M+H]^+$

(5R,8S,11R,13S,14S,17R)-11-(4-(dimethylamino)phenyl)-17-ethynyl-13-methyl-1,2,6,7,8,11,12,13,14,15,16,17-dodecahydrospiro[cyclopenta[a]phenanthrene-3,2'-[1,3]dioxolane]-5,17(4H)-diol (22)

Trimethylsilylacetylene (424 μl, 2.7 mmol) was dissolved in anhydrous THF (10 ml) and cooled to -78°C. To the solution 2.5M *n*BuLi (1.9 ml) was added dropwise and following the complete addition the solution was allowed to stir for 1 h. After 1 h **4** (0.3 g, 0.66 mmol) in THF (0.5 mL) was added dropwise and the reaction was allowed to warm to rt and was stirred for 18 h. Upon completion the reaction was quenched with $NH_4Cl_{(aq)}$ and the crude mixture was extracted with EtOAc (2 × 10 ml). The organic fractions were collected, combined and reduced *in vacuo.* Boiling ether was added to the crude material and the compound was precipitated from ether through the dropwise addition of Pet. Spirits. The solid precipitate was collected by vacuum filtration to give **30** (0.22 g, 0.39 mmol, 59%). **30** (0.02 g, 0.04 mmol) was dissolved in THF (1 ml) and 1 M TBAF in THF (90 μl, 0.09 mmol) was added. The reaction was stirred for 2 h the quenched with $NaHCO_{3(aq)}$ (1 ml) and taken up in CH_2Cl_2 (5 ml). The organic layer was collected, then dried over anhydrous $MgSO_4$, filtered and evaporated *in vacuo*. Flash chromatography of the crude using eluent toluene: acetone (10: 1) delivered the title compound (0.012 g, 0.038 mmol, 96%). ^{1}H−NMR (400 MHz; $CDCl_3$): δ 7.06 (d, *J* = 8.3 Hz, 2H), 6.65 (d, *J* = 8.9 Hz, 2H), 4.37 (d, *J* = 1.2 Hz, 1H), 4.26 (d, *J* = 6.9 Hz, 1H), 4.04-3.90 (m, 4H), 2.91 (s, 6H),

2.50-2.45 (m, 1H), 2.37-2.20 (m, 5H), 2.14-2.10 (m, 1H), 2.05-1.89 (m, 3H), 1.83-1.68 (m, 4H), 1.65-1.49 (m, 3H), 1.36-1.25 (m, 3H), 0.50 (s, 3H). ^{13}C–NMR (101 MHz; $CDCl_3$): δ 148.5, 134.7, 134.3, 134.0, 129.2, 128.4, 127.8, 125.4, 112.8, 109.0, 87.3, 80.3, 74.7, 70.3, 64.8, 64.2, 49.8, 47.6, 46.8, 40.9, 39.03, 39.00, 38.65, 38.57, 38.39, 35.3, 24.2, 23.7, 23.4, 13.6. LCMS: 460.3 $[M-17]^+$

(5*R*,8*S*,11*R*,13*S*,14*S*)-11-(4-(dimethylamino)phenyl)-13-methyl-17-(2-((4-nitrophenyl)sulfinyl)vinylidene)-1,2,6,7,8,11,12,13,14,15,16,17-dodecahydrospiro[cyclopenta[*a*]phenanthrene-3,2'-[1,3]dioxolan]-5(4*H*)-ol (23)

22 (0.15 g, 0.31 mmol) was dissolved in anhydrous THF (6 ml), TEA (350 µl, 2.51 mmol) was then added and the reaction was cooled to −78°C. 4-nitrophenylsulfenylchloride (0.2 g, 1.07 mmol) was added drop wise maintaining the temperature at −78°C for 2 h. The reaction was allowed to slowly warm to rt after 2 h and stir for 18 h. The reaction was then quenched with H_2O. The aqueous layer was then extracted with CH_2Cl_2 (2 × 10 ml). The organic layer was then dried over anhydrous $MgSO_4$, filtered and evaporated *in vacuo.* Crude product was isolated using flash chromatography using eluent EtOAc: Pet. spirits (2: 1) to give the title compound as a set of stereoisomers in a ratio of (60*:40$^\#$), which were not separable by flash chromatography (0.09 g, 0.14 mmol, 45%). ^{1}H–NMR (400 MHz; $CDCl_3$): δ 8.41* (d, *J* = 9.0 Hz, 2H), 8.36$^\#$ (d, *J* = 9.0 Hz, 2H), 7.80$^{*\#}$ (dd, *J* = 9.0, 8.0 Hz, 4H), 7.01$^\#$ (d, *J* = 8.5 Hz, 2H), 6.97* (d, *J* = 8.5 Hz, 2H), 6.71* (d, *J* = 8.9 Hz, 2H), 6.67$^\#$ (d, *J* = 8.9 Hz, 1H), 6.14-6.13$^{*\#}$ (m, 2H), 4.35$^{*\#}$ (s, 2H), 4.21-4.14$^{*\#}$ (m, 3H), 4.02-3.89$^{*\#}$ (m, 8H), 2.96* (s, 6H), 2.93$^\#$ (s, 6H), 2.68-1.42$^{*\#}$ (m, 37H), 0.63* (s, 3H), 0.62$^\#$ (s, 2H). ^{13}C–NMR (101 MHz; $CDCl_3$): δ 197.1, 149.4, 134.9, 134.6, 134.0, 133.7, 127.8, 125.49, 125.41, 124.42, 124.39, 123.3, 122.7, 112.81, 112.67, 108.88, 108.83, 105.0, 104.5, 70.13, 70.10, 64.8, 64.2, 54.8, 54.5, 47.51, 47.39, 47.28, 42.6, 41.8, 40.86, 40.80, 38.98, 38.93, 38.6, 38.4, 35.20, 35.13, 28.1, 27.7, 25.7, 24.7, 23.40, 23.36, 20.00, 19.86.

LCMS: FAILED

(8*S*,11*R*,13*S*,14*S*,17*R*)-17-acetyl-11-(4-(dimethylamino)phenyl)-17-hydroxy-13-methyl-1,2,6,7,8,11,12,13,14,15,16,17-dodecahydro-3*H*-cyclopenta[*a*]phenanthren-3-one (25)

Sodium Methoxide (0.008 g, 0.15 mmol) in methanol (25% w/w) was diluted with anhydrous methanol (2 ml) followed by the addition of **23** and the resultant reaction was heated to 70°C for 1 h. After 1 h trimethyl phosphite (0.03 g, 0.21 mmol) was added and the reaction was allowed to stir for an additional 2 h at 70°C. After 2 h the reaction was allowed to cool to rt and water was added to the reaction (10 ml). The aqueous solution was extracted with CH_2Cl_2 (4 × 10 ml). The organic layers were collected and subsequently washed with brine (1 × 10 ml), collected and then dried over anhydrous $MgSO_4$, filtered and evaporated *in vacuo.* Crude product was isolated using flash chromatography using eluent EtOAc: Pet. spirits (2: 1) to give the intermediate compound (0.02 g, 0.039 mmol, 28%), which was carried to the next step without characterisation. The intermediate was subsequently dissolved in methanol (1 ml) followed by the addition of 1 M HCl (20 μl) at rt. The reaction was stirred for 45 min at rt then quenched by the addition of H_2O and neutralized by the addition of $NaHCO_3$. The aqueous solution was extracted with CH_2Cl_2 (2 × 10 ml). The organic layers were collected and subsequently washed with brine (1 × 10 ml), collected and then dried over anhydrous $MgSO_4$, filtered and evaporated *in vacuo.* Crude LCMS showed the conversion to the methyl ketone intermediate along with partial elimination of the hydroxyl group therefore without purification the compound was carried to the next stage. The crude material was dissolved in in 70% AcOH (2 ml) and heated to 50°C for 2 h. The crude reaction was then diluted by the addition of H_2O and neutralized to pH 7 by the drop wise addition of 2 M NaOH. The aqueous layer was then extracted with CH_2Cl_2 (2 × 10 ml). The organic layer was then dried over anhydrous $MgSO_4$, filtered and evaporated *in vacuo.* Crude product was isolated using flash chromatography using eluent toluene: acetone (10:

1) to give the title compound (0.013 g, 0.03 mmol, 77%) ^1H−NMR (400 MHz; $CDCl_3$): δ 6.98 (d, *J* = 8.3 Hz, 2H), 6.63 (d, *J* = 8.9 Hz, 2H), 5.75 (s, 1H), 4.37-4.35 (m, 1H), 3.04 (s, 1H), 2.90 (s, 6H), 2.76-2.71 (m, 1H), 2.67-2.62 (m, 1H), 2.60-2.57 (m, 2H), 2.50-2.49 (m, 1H), 2.43-2.31 (m, 4H), 2.25 (s, 3H), 2.06-1.99 (m, 3H), 1.94-1.86 (m, 1H), 1.68-1.61 (m, 1H), 1.57-1.39 (m, 2H), 0.47 (s, 3H). ^{13}C−NMR (101 MHz; CDCl3): δ 212.0, 199.8, 156.9, 148.7, 146.2, 131.9, 129.3, 127.6, 122.9, 112.8, 89.7, 50.0, 48.9, 40.7, 39.4, 38.3, 37.0, 36.0, 33.4, 31.2, 28.23, 28.04, 26.0, 24.5, 17.1. LCMS: 434.3 $[M+H]^+$

(8*S*,11*R*,13*S*,14*S*,17*R*)-17-acetyl-11-(4-(dimethylamino)phenyl)-13-methyl-3-oxo-2,3,6,7,8,11,12,13,14,15,16,17-dodecahydro-1*H*-cyclopenta[*a*]phenanthren-17-yl acetate (21)

AcOH was added to a solution of CH_2Cl_2 and stirred for 30 min at rt. Catalytic *p*-toluenesulphonic acid was added and the reaction mixture was cooled to 0°C. **25** (0.01 g, 0.023) dissolved in CH_2Cl_2 was the added and the reaction mixture was stirred at 0°C for 30 min. The reaction was monitored by TLC showing the complete reaction of starting material after 1 h. The reaction was subsequently neutralised by the addition of K_2CO_3(aq). Aqueous was extracted with CH_2Cl_2 (2 × 10 ml). The organic layers were then dried over anhydrous $MgSO_4$, filtered and evaporated *in vacuo.* Crude product was isolated using flash chromatography using eluent toluene: acetone (10: 1) to give the title compound **21** (0.003, 0.006, 25%). ^1H−NMR (400 MHz; $CDCl_3$): δ 7.00-6.95 (m, 2H), 6.66-6.61 (m, 2H), 5.78-5.77 (m, 1H), 4.40-4.37 (m, 1H), 2.92-2.89 (m, 6H), 2.81-2.73 (m, 1H), 2.62-2.48 (m, 5H), 2.45-2.31 (m, 4H), 2.15-2.11 (m, 3H), 2.11-2.08 (m, 3H), 2.07-1.93 (m, 3H), 1.86-1.76 (m, 2H), 1.43-1.31 (m, 1H), 0.37-0.33 (m, 3H). ^{13}C−NMR (101 MHz; $CDCl_3$): δ 203.8, 199.5, 170.7, 156.6, 148.9, 145.7, 131.6, 129.5, 127.5, 123.2, 112.9, 96.4, 51.1, 47.3, 40.7, 39.6, 38.5, 37.04, 36.95, 31.2, 30.5, 28.1, 27.0, 26.0, 24.4, 21.4, 15.8. LCMS: 476.0 $[M+H]^+$

(8*S*,11*R*,13*S*,14*S*,17*R*)-11-(4-(dimethylamino)phenyl)-13-methyl-3-oxo-2,3,6,7,8,11,12,13,14,15,16,17-dodecahydro-1*H*-cyclopenta[*a*]phenanthrene-17-carbonitrile (26)

4 (0.1 g, 0.22 mmol) was dissolved in anhydrous DME (6 ml) and *t*BuOH (0.2 ml) and the mixture was stirred for 10 min, after which *t*BuOK (0.25 g, 2.2 mmol) was added in one portion and the reaction was stirred for 5 min. Tosylmethylisocyanide (0.09 g, 0.44 mmol) in anhydrous DME (1.1 ml) and *t*BuOH (122 µl) was added to the reaction over 1 h. After 1 h the reaction was quenched with $NH_4Cl_{(aq)}$ and neutralized to pH 7 with the drop wise addition of 1M HCl. The aqueous layer was then extracted with EtOAc (2 × 10 ml) and brine (10 ml). The organic layer was then dried over anhydrous $MgSO_4$, filtered and evaporated *in vacuo*. Crude product was isolated using flash chromatography using eluent Pet. spirits: EtOAc (1: 1) to give the intermediate diketal compound, which was separated as separate epimers. Trans epimer (0.039 g, 0.085 mmol, 39%) : ^{1}H−NMR (400 MHz; $CDCl_3$): δ 7.04 (d, *J* = 8.4 Hz, 2H), 6.65 (d, *J* = 8.8 Hz, 2H), 4.37 (d, *J* = 1.1 Hz, 1H), 4.22 (dd, *J* = 6.7, 0.3 Hz, 1H), 4.05-3.89 (m, 4H), 2.91 (s, 6H), 2.57 (dd, *J* = 13.1, 1.2 Hz, 1H), 2.49-2.44 (m, 1H), 2.36-2.31 (m, 1H), 2.28-1.20 (m, 16H), 0.56 (s, 3H). ^{13}C−NMR (101 MHz; $CDCl_3$): δ 148.6, 134.7, 133.8, 127.7, 121.3, 112.8, 108.9, 70.1, 64.8, 64.2, 53.9, 47.5, 44.5, 43.1, 40.96, 40.81, 38.7, 38.4, 38.1, 35.2, 26.6, 25.2, 24.6, 23.4, 15.2.

The Trans 17-carbonitrile intermediate (0.03 g, 0.06 mmol) was dissolved in 70% AcOH (2 ml) and heated to 50°C for 2 h. The crude reaction was then diluted by the addition of H_2O and neutralized to pH 7 by the drop wise addition of 2M NaOH. The aqueous layer was then extracted with CH_2Cl_2 (2 × 10 ml). The organic layer was then dried over anhydrous $MgSO_4$, filtered and evaporated *in vacuo* to give the title compound without the requirement of flash (0.008 g, 0.02 mmol, 33%). ^{1}H−NMR (400 MHz; $CDCl_3$): δ 7.01 (dd, *J* = 8.9, 0.7 Hz, 2H), 6.66 (d, *J* = 8.9 Hz, 2H), 5.77 (s, 1H), 4.42-4.40 (m, 1H), 2.92 (s, 6H), 2.79-2.74 (m, 1H), 2.60-2.56

(m, 3H), 2.47-1.27 (m, 13H), 0.53 (s, 3H). ^{13}C−NMR (101 MHz; $CDCl_3$): δ 199.7, 156.3, 148.8, 145.0, 131.3, 129.8, 127.6, 123.3, 122.0, 112.9, 51.7, 44.4, 40.9, 40.71, 40.53, 39.6, 38.7, 37.0, 31.1, 28.1, 27.3, 26.1, 25.4, 19.2. LCMS: 401.3 $[M+H]^+$

(8*S*,11*R*,13*S*,14*S*,17*S*)-11-(4-(dimethylamino)phenyl)-13-methyl-3-oxo-2,3,6,7,8,11,12,13,14,15,16,17-dodecahydro-1*H*-cyclopenta[*a*]phenanthrene-17-carbonitrile (27)

4 (0.1 g, 0.22 mmol) was dissolved in anhydrous DME (6 ml) and *t*BuOH (0.2 ml) and the mixture was stirred for 10 min, after which *t*BuOK (0.25 g, 2.2 mmol) was added in one portion and the reaction was stirred for 5 min. Tosylmethylisocyanide (0.09 g, 0.44 mmol) in anhydrous DME (1.1 ml) and *t*BuOH (122 µl) was added to the reaction over 1 h. After 1 h the reaction was quenched with $NH_4Cl_{(aq)}$ and neutralized to pH 7 with the drop wise addition of 1M HCl. The aqueous layer was then extracted with EtOAc (2 × 10 ml) and brine (10 ml). The organic layer was then dried over anhydrous $MgSO_4$, filtered and evaporated *in vacuo*. Crude product was isolated using flash chromatography using eluent Pet. spirits: EtOAc (1: 1) to give the intermediate diketal compound, which was separated as separate epimers. Cis isomer (0.046 g, 0.10 mmol, 47%): ^{1}H−NMR (400 MHz; $CDCl_3$): δ 7.04 (d, *J* = 8.3 Hz, 2H), 6.64 (d, *J* = 8.9 Hz, 2H), 4.31-4.30 (m, 1H), 4.27 (s, 1H), 4.04-3.92 (m, 4H), 2.91 (s, 6H), 2.53-2.44 (m, 2H), 2.40-2.28 (m, 3H), 2.16-1.24 (m, 14H), 0.45 (s, 3H). ^{13}C−NMR (101 MHz; $CDCl_3$): δ 148.5, 134.7, 133.65, 133.47, 127.7, 122.1, 112.8, 108.9, 70.0, 64.8, 64.2, 51.7, 47.6, 44.2, 40.96, 40.81, 40.5, 38.63, 38.52, 38.3, 35.2, 27.4, 25.8, 24.8, 23.4, 19.1. Cis epimer (0.035 g, 0.08 mmol) was dissolved in 70% AcOH (2 ml) and heated to 50°C for 2 h. The crude reaction was then diluted by the addition of H_2O and neutralized to pH 7 by the drop wise addition of 2 M NaOH. The aqueous layer was then extracted with CH_2Cl_2 (2 × 10 ml). The organic layer was then dried over anhydrous $MgSO_4$, filtered and evaporated *in vacuo* to give the title compound without the

requirement of flash (0.008 g, 0.02 mmol, 25%). ^{1}H−NMR (400 MHz; $CDCl_3$): δ 7.01 (d, *J* = 8.3 Hz, 2H), 6.67 (d, *J* = 8.9 Hz, 2H), 5.76 (s, 1H), 4.34 (dd, *J* = 6.7, 0.3 Hz, 1H), 2.92 (s, 6H), 2.77-1.27 (m, 17H), 0.63 (s, 3H). ^{13}C− NMR (101 MHz; $CDCl_3$): δ 199.6, 156.3, 148.9, 145.3, 130.8, 129.8, 127.6, 123.3, 121.1, 112.9, 53.9, 44.7, 43.2, 41.0, 40.7, 39.7, 38.8, 37.0, 31.1, 28.0, 26.5, 26.0, 24.9, 15.4. LCMS: 401.3 $[M+H]^+$

(8*S*,11*R*,13*S*,14*S*)-11-(4-(dimethylamino)phenyl)-17-hydroxy-13-methyl-17-(trifluoromethyl)-1,2,6,7,8,11,12,13,14,15,16,17-dodecahydro-3*H*-cyclopenta[*a*]phenanthren-3-one (28) & (29)

4 (0.17 g, 0.38 mmol) was dissolved in anhydrous THF (1.3 ml) and CF_3SiMe_3 (271 µl, 1.7 mmol) was added to the solution and the mixture was cooled to −5°C. Me_4NF (0.009 g, 0.09 mmol) was then added and stirred at −5°C for 1 h, then stirred for 4 h at rt. The reaction was subsequently quenched with $NaHCO_3$ and extracted with EtOAc (2 × 20 ml). The organic layer was then dried over anhydrous $MgSO_4$, filtered and evaporated *in vacuo*. Crude product was isolated using flash chromatography using eluent: toluene: acetone (13: 1) to give the ketal derivative, which was separated as separate epimers. Epimer 1 (0.045 g, 0.086 mmol, 23%) and Epimer 2 (0.041, 0.079 mmol, 21%), because of instability of the ketal protected derivative the reaction was carried over to the next step. Each epimer was reacted separately. Epimer 1 (0.45 g, 0.086 mmol) and Epimer 2 (0.041 g, 0.079 mmol) were individually dissolved in 70% AcOH (2 ml) and heated to 50°C for 2 h. The crude reaction was then diluted by the addition of H_2O and neutralized to pH 7 by the drop wise addition of 2 M NaOH. The aqueous layer was then extracted with CH_2Cl_2 (2 × 10 ml). The organic layer was then dried over anhydrous $MgSO_4$, filtered and evaporated *in vacuo*. Crude product was isolated using flash chromatography using eluent toluene: acetone (9: 1) to give the title compound epimer 1 (0.012 g, 0.035 mmol, 30%) and epimer 2 (0.015 g, 0.03 mmol, 41%). Epimer 1: ^{1}H−NMR (400 MHz;

$CDCl_3$): δ 7.02 (d, *J* = 8.3 Hz, 2H), 6.66 (d, *J* = 8.8 Hz, 2H), 5.78 (s, 1H), 4.35 (d, *J* = 6.9 Hz, 1H), 2.91 (s, 6H), 2.78-2.72 (m, 1H), 2.68-2.60 (m, 3H), 2.53-2.33 (m, 6H), 2.16-1.99 (m, 3H), 1.90-1.85 (m, 1H), 1.62-1.51 (m, 3H), 0.59 (s, 3H). ^{13}C−NMR (101 MHz; $CDCl_3$): δ 219.4, 199.7, 156.4, 148.8, 145.7, 131.4, 129.9, 127.6, 123.3, 112.9, 50.8, 47.8, 40.7, 39.5, 38.1, 37.7, 37.0, 35.6, 31.0, 26.9, 26.0, 22.0, 14.6. LCMS: 460.0 $[M+H]^+$

Epimer 2: ^{1}H−NMR (400 MHz; $CDCl_3$): δ 7.01 (d, *J* = 8.4 Hz, 2H), 6.66 (d, *J* = 8.8 Hz, 2H), 5.76 (s, 1H), 4.37-4.35 (m, 1H), 2.92 (s, 6H), 2.77-2.71 (m, 1H), 2.60-2.50 (m, 3H), 2.45-2.28 (m, 5H), 2.18-2.13 (m, 2H), 2.05-2.01 (m, 1H), 1.82-1.75 (m, 3H), 1.50-1.42 (m, 2H), 0.64 (s, 3H). ^{13}C−NMR (101 MHz; $CDCl_3$): δ 199.8, 156.6, 148.7, 145.4, 131.7, 129.7, 127.5, 123.1, 112.9, 95.1, 84.7, 51.0, 47.3, 40.8, 39.8, 39.3, 38.0, 37.0, 33.3, 31.2, 27.8, 25.9, 24.3, 16.1. LCMS: 460.0 $[M+H]^+$

(5*R*,8*S*,11*R*,13*S*,14*S*,17*S*)-11-(4-(dimethylamino)phenyl)-13-methyl-17-((trimethylsilyl)ethynyl)-1,2,6,7,8,11,12,13,14,15,16,17-dodecahydrospiro[cyclopenta[*a*]phenanthrene-3,2'-[1,3]dioxolane]-5,17(4*H*)-diol (30)

Trimethylsilylacetylene (424 µl, 2.7 mmol) was added to anhydrous THF (10 ml) and cooled to -78°C, after which 2.5 M *n*BuLi (1.9 ml) was added dropwise and the solution stirred for 1 h. After 1 h **4** (0.3 g, 0.66 mmol) in THF (0.5 mL) was added dropwise and the reaction was allowed to warm to rt and was stirred for 18 h. Upon completion the reaction was quenched with $NH_4Cl_{(aq)}$ and the crude mixture was extracted with EtOAc (2 × 10 ml). The organic fractions were collected, combined and reduced *in vacuo*. Boiling ether was added to the crude material and the compound was precipitated from ether through the dropwise addition of Pet. Spirits. The solid precipitate was collected by vacuum filtration to give the title compound (0.22 g, 0.39 mmol, 59%). ^{1}H−NMR (400 MHz; $CDCl_3$): δ 7.06 (d, *J* = 8.5 Hz, 2H), 6.64 (d, *J* = 8.9 Hz, 2H), 4.41 (d, *J* = 0.9 Hz, 1H), 4.24 (dt, *J* = 7.2, 0.2 Hz, 1H), 4.04-3.90 (m, 4H), 2.90 (s, 6H), 2.49-

2.44 (m, 1H), 2.36-0.86 (m, 18H), 0.48 (s, 3H), 0.20 (s, 9H). ^{13}C−NMR (101 MHz; $CDCl_3$): δ 148.4, 134.8, 133.8, 127.9, 112.7, 109, 90.8, 80.7, 70.4, 64.9, 64.2, 49.8, 47.7, 46.8, 40.9, 39.5, 39.3, 39.1, 38.8, 38.6, 35.3, 24.3, 23.9, 23.4, 13.6, 0.3. LCMS: 550.3 $[M+H]^+$

(5*R*,8*S*,11*R*,13*S*,14*S*,17*S*)-11-(4-(dimethylamino)phenyl)-13-methyl-17-((triethylsilyl)ethynyl)-1,2,6,7,8,11,12,13,14,15,16,17-dodecahydrospiro[cyclopenta[*a*]phenanthrene-3,2'-[1,3]dioxolane]-5,17(4*H*)-diol (31)

Triethylsilylacetylene (358 µl, 2,0 mmol) was added to anhydrous THF (5 ml) and cooled to -78°C, after which 2.5 M *n*BuLi (1.6 ml) was added dropwise, and the solution stirred for 1 h. After 1 h **4** (0.23 g, 0.5 mmol) in THF (1.0 ml) was added dropwise and the reaction was allowed to warm to rt and was stirred for 18 h. Upon completion the reaction was quenched with $NH_4Cl_{(aq)}$ and the crude mixture was extracted with EtOAc (2 × 10 mL). The organic fractions were collected, combined and reduced *in vacuo*. Flash Chromatography using eluent Pet. Spirits: EtOAc (3: 1) delivered the title compound (0.08 g, 0.14 mmol, 28%). ^{1}H−NMR (400 MHz; $CDCl_3$): δ 7.06 (d, J = 8.5 Hz, 2H), 6.64 (d, J = 8.8 Hz, 2H), 4.27-4.23 (m, 2H), 4.04-3.90 (m, 4H), 2.90 (s, 6H), 2.49-2.44 (m, 1H), 2.37-1.25 (m, 18H), 1.00 (t, J = 7.9 Hz, 9H), 0.62 (q, J = 8.0 Hz, 6H), 0.48 (s, 3H). ^{13}C−NMR (101 MHz; $CDCl_3$): δ 148.4, 134.72, 134.64, 133.9, 127.8, 112.7, 110.7, 109.0, 88.2, 80.7, 70.3, 64.8, 64.1, 50.1, 47.7, 46.8, 40.9, 39.5, 39.1, 38.9, 38.7, 38.5, 35.3, 24.2, 23.8, 23.3, 13.7, 7.7, 4.6. LCMS: 592.5 $[M+H]^+$

(5*R*,8*S*,11*R*,13*S*,14*S*,17*S*)-11-(4-(dimethylamino)phenyl)-13-methyl-17-((triisopropylsilyl)ethynyl)-1,2,6,7,8,11,12,13,14,15,16,17-dodecahydrospiro[cyclopenta[*a*]phenanthrene-3,2'-[1,3]dioxolane]-5,17(4*H*)-diol (32)

Triisopropylsilylacetylene (157 µL, 0.71 mmol) was dissolved in anhydrous THF (3 mL) and cooled to -78°C. To the solution 2.5M *n*BuLi (570 µL) was added dropwise and following the

complete addition the solution was allowed to stir for 1 h. After 1 h **4** (0.08 g, 0.18 mmol) in THF (0.5 mL) was added dropwise and the reaction was allowed to warm to rt and was stirred for 18 h. Upon completion the reaction was quenched with $NH_4Cl_{(aq)}$ and the crude mixture was extracted with EtOAc (2 × 10 mL). The organic fractions were collected, combined and reduced *in vacuo*. Flash Chromatography using eluent Pet. Spirits: EtOAc (2: 1) delivered the title compound (0.017 g, 0.03 mmol, 17%). ^{1}H−NMR (400 MHz; $CDCl_3$): δ 7.07 (d, *J* = 8.4 Hz, 2H), 6.64 (d, *J* = 8.9 Hz, 2H), 4.24-4.22 (m, 1H), 4.11 (s, 1H), 4.04-3.88 (m, 4H), 2.91 (s, 6H), 2.50-2.44 (m, 1H), 2.42-2.37 (m, 1H), 2.33-2.21 (m, 3H), 2.13-2.09 (m, 1H), 2.04-1.91 (m, 4H), 1.81-1.20 (m, 12H), 1.07 (s, 18H), 0.49 (s, 3H). ^{13}C−NMR (101 MHz; $CDCl_3$): δ 148.4, 134.7, 134.4, 134.0, 127.8, 112.7, 111.4, 109.0, 87.1, 80.8, 70.3, 64.7, 64.1, 50.3, 47.6, 46.9, 40.9, 39.5, 38.78, 38.62, 38.54, 38.3, 35.3, 24.17, 23.98, 23.7, 23.2, 18.8, 13.8, 11.4. LCMS: 634.5 $[M+H]^+$

(8*S*,11*R*,13*S*,14*S*,17*S*)-11-(4-(dimethylamino)phenyl)-17-hydroxy-13-methyl-17-((trimethylsilyl)ethynyl)-1,2,6,7,8,11,12,13,14,15,16,17-dodecahydro-3*H*-cyclopenta[*a*]phenanthren-3-one (33)

30 (0.06 g, 0.10 mmol) was dissolved in 70% AcOH (2 ml) and heated to 50°C for 2 h, followed by dilution with H_2O and neutralization to pH 7 by the dropwise addition of 2 M NaOH. The aqueous layer was then extracted with CH_2Cl_2 (2 × 10 ml). The organic layer was then dried over anhydrous $MgSO_4$, filtered and evaporated *in vacuo*. Flash chromatography of the crude using eluent toluene: acetone (11:1) delivered the title compound (0.02 g, 0.04 mmol, 39%). ^{1}H−NMR (400 MHz; $CDCl_3$): δ 7.02 (d, *J* = 8.4 Hz, 2H), 6.66 (d, *J* = 8.9 Hz, 2H), 5.76 (s, 1H), 4.36 (d, *J* = 5.9 Hz, 1H), 2.91 (s, 6H), 2.82-1.32 (m, 8H), 0.55 (s, 3H), 0.19 (s, 9H). ^{13}C−NMR (101 MHz; $CDCl_3$): δ 199.9, 157.1, 148.7, 146.8, 132.1, 129.3, 127.7, 122.9, 112.9, 109.1, 91.0, 80.4, 50.2, 47.1, 40.8, 39.7, 39.2, 39.01, 38.94, 37.1, 31.2, 27.5, 26.0, 23.5, 13.8, 0.2. LCMS: 488.3 $[M+H]^+$

(8*S*,11*R*,13*S*,14*S*,17*S*)-11-(4-(dimethylamino)phenyl)-17-hydroxy-13-methyl-17-((triethylsilyl)ethynyl)-1,2,6,7,8,11,12,13,14,15,16,17-dodecahydro-3*H*-cyclopenta[*a*]phenanthren-3-one (34)

31 (0.08 g, 0.14 mmol) was dissolved in 70% AcOH (2 ml) and heated to 50°C for 2 h, followed by dilution with H_2O and neutralization to pH 7 by the dropwise addition of 2 M NaOH. The aqueous layer was then extracted with CH_2Cl_2 (2 × 10 ml). The organic layer was then dried over anhydrous $MgSO_4$, filtered and evaporated *in vacuo*. Flash chromatography of the crude using eluent toluene: acetone (11: 1) delivered the title compound (0.02 g, 0.04 mmol, 32%). ^{1}H−NMR (400 MHz; $CDCl_3$): δ 7.03 (d, *J* = 8.3 Hz, 2H), 6.66 (d, *J* = 8.9 Hz, 2H), 5.76 (s, 1H), 4.35 (d, *J* = 6.8 Hz, 1H), 2.92 (s, 6H), 2.77-2.69 (m, 1H), 2.55 (dd, *J* = 7.8, 4.5 Hz, 2H), 2.51-2.24 (m, 7H), 2.05-1.91 (m, 3H), 1.87-1.70 (m, 2H), 1.50-1.33 (m, 2H), 0.99 (t, *J* = 7.9 Hz, 9H), 0.61 (q, *J* = 8.0 Hz, 6H), 0.56 (s, 3H). ^{13}C−NMR (101 MHz; $CDCl_3$): δ 199.9, 157.2, 148.7, 146.9, 131.9, 129.1, 127.6, 122.8, 112.8, 110.5, 88.3, 80.4, 50.3, 47.1, 40.8, 39.6, 39.1, 38.71, 38.58, 37.2, 30.9, 27.3, 26.0, 23.4, 13.9, 7.7, 4.6. LCMS: 530.4 $[M+H]^+$

(8*S*,11*R*,13*S*,14*S*,17*S*)-11-(4-(dimethylamino)phenyl)-17-hydroxy-13-methyl-17-((triisopropylsilyl)ethynyl)-1,2,6,7,8,11,12,13,14,15,16,17-dodecahydro-3*H*-cyclopenta[*a*]phenanthren-3-one (35)

32 (0.04 g, 0.06 mmol) was dissolved in 70% AcOH (2 ml) and heated to 50°C for 2 h, followed by dilution with H_2O and neutralization to pH 7 by the dropwise addition of 2 M NaOH. The aqueous layer was then extracted with CH_2Cl_2 (2 × 10 ml). The organic layer was then dried over anhydrous $MgSO_4$, filtered and evaporated *in vacuo*. Flash chromatography of the crude using eluent toluene: acetone (11: 1) delivered the title compound (0.01 g, 0.02 mmol, 29%). ^{1}H−NMR (400 MHz; $CDCl_3$): δ 7.04 (d, *J* = 8.3 Hz, 2H), 6.67 (d, *J* = 8.9 Hz, 2H), 5.75 (s, 1H), 4.35-4.33 (m, 1H), 2.92 (s, 6H), 2.75-2.69 (m, 1H), 2.55-2.50 (m, 2H), 2.49-2.42 (m, 2H), 2.39-2.34 (m,

3H), 2.32-2.25 (m, 2H), 2.03-1.89 (m, 3H), 1.89-1.86 (m, 1H), 1.77-1.68 (m, 1H), 1.61 (s, 3H), 1.49-1.32 (m, 2H), 1.07 (s, 18H), 0.57 (s, 3H). ^{13}C−NMR (101 MHz; $CDCl_3$): δ 199.9, 157.2, 148.7, 147.1, 131.8, 129.0, 127.6, 122.8, 112.8, 111.1, 87.3, 80.5, 50.3, 47.3, 40.8, 39.5, 39.2, 38.37, 38.33, 37.3, 30.6, 27.2, 26.1, 23.2, 18.8, 13.9, 11.3. LCMS: 572.5 $[M+H]^+$

(5*R*,8*S*,11*R*,13*S*,14*S*,17*S*)-11-(4-(dimethylamino)phenyl)-13-methyl-17-(phenylethynyl)-1,2,6,7,8,11,12,13,14,15,16,17-dodecahydrospiro[cyclopenta[*a*]phenanthrene-3,2'-[1,3]dioxolane]-5,17(4*H*)-diol (36)

22 (0.08 g, 0.17 mmol) was dissolved in anhydrous THF (1.5 ml), followed by the addition of CuI (0.003 g, 0.003 mmol), Et_3N (116 μl, 0.84 mmol) and iodobenzene (34 μl, 0.27 mmol). The solution was then degassed for a period of 30 min following which $PdCl_2(PPh_3)_2$ (0.002 g, 0.003 mmol) was added and the mixture further degassed for 20 min and then subsequently heated at 80°C for 24 h. The reaction was then quenched by the addition of $NH_4Cl_{(aq)}$ and extracted with EtOAc (2 × 10 ml). The organic layers were collected and dried over anhydrous $MgSO_4$, filtered and evaporated *in vacuo*. Flash chromatography of the crude using eluent toluene: acetone (20: 1) delivered the title compound (0.073 g, 0.13 mmol, 79%). ^{1}H−NMR (400 MHz; $CDCl_3$): δ 7.47-7.45 (m, 2H), 7.33-7.30 (m, 3H), 7.10 (d, *J* = 8.6 Hz, 2H), 6.73 (s, 2H), 4.32 (s, 1H), 4.28 (d, *J* = 7.1 Hz, 1H), 4.03-3.90 (m, 4H), 2.93 (s, 6H), 2.48-1.26 (m, 19H), 0.54 (s, 3H). 13−C NMR (101 MHz; $CDCl_3$): δ 134.6, 134.2, 131.9, 128.3, 128.0, 123.0, 113.3, 109.0, 92.6, 86.6, 80.8, 70.3, 64.8, 64.1, 50.1, 47.7, 47.3, 41.3, 39.41, 39.33, 39.31, 38.8, 38.6, 35.2, 24.3, 24.0, 23.4, 13.8. LCMS: 554.0 $[M+H]^+$

(5*R*,8*S*,11*R*,13*S*,14*S*,17*S*)-11-(4-(dimethylamino)phenyl)-17-((4-methoxyphenyl)ethynyl)-13-methyl-1,2,6,7,8,11,12,13,14,15,16,17-dodecahydrospiro[cyclopenta[*a*]phenanthrene-3,2'-[1,3]dioxolane]-5,17(4*H*)-diol (37)

22 (0.08 g, 0.17 mmol) was dissolved in anhydrous THF (2.5 ml), followed by the addition of CuI (0.003 g, 0.013 mmol), Et_3N (116 µl, 0.84 mmol) and 4-bromoanisole (0.05 g, 0.27 mmol). The solution was then degassed for a period of 30 min following which $PdCl_2(PPh_3)_2$ (0.002 g, 0.003 mmol) was added and the mixture further degassed for 20 min and then subsequently heated at 80°C for 24 h. The reaction was then quenched by the addition of $NH_4Cl_{(aq)}$ and extracted with EtOAc (2 × 10 ml). The organic layers were collected and dried over anhydrous $MgSO_4$, filtered and evaporated *in vacuo*. Flash chromatography of the crude using eluent toluene: acetone (6: 1) delivered the title compound (0.013 g, 0.022 mmol, 13%). ^{1}H−NMR (400 MHz; $CDCl_3$): δ 7.39 (d, *J* = 8.9 Hz, 2H), 7.08 (d, *J* = 8.6 Hz, 2H), 6.84 (d, *J* = 8.9 Hz, 2H), 6.67 (d, *J* = 8.5 Hz, 2H), 4.33 (s, 1H), 4.27 (d, *J* = 6.8 Hz, 1H), 4.02-3.92 (m, 4H), 3.82 (s, 3H), 2.91 (s, 6H), 2.49-2.25 (m, 5H), 2.18-2.10 (m, 1H), 2.04-1.97 (m, 4H), 1.84-1.49 (m, 7H), 1.39-1.27 (m, 2H), 0.54 (s, 3H). ^{13}C− NMR (101 MHz; $CDCl_3$): δ 159.7, 134.7, 134.0, 133.3, 127.9, 115.2, 114.0, 112.9, 109.0, 91.2, 86.4, 80.8, 70.3, 64.7, 64.1, 55.4, 50.1, 47.7, 47.2, 41.0, 39.40, 39.33, 39.26, 38.8, 38.6, 35.2, 24.3, 24.0, 23.4, 13.8. LCMS: 584.0 $[M+H]^+$

(8*S*,11*R*,13*S*,14*S*,17*S*)-11-(4-(dimethylamino)phenyl)-17-hydroxy-13-methyl-17-(phenylethynyl)-1,2,6,7,8,11,12,13,14,15,16,17-dodecahydro-3*H*-cyclopenta[*a*]phenanthren-3-one (38)

36 (0.06 g, 0.11 mmol) was dissolved in 70% AcOH (2 ml) and heated to 50°C for 2 h. The crude reaction was then diluted by the addition of H_2O and neutralized to pH 7 by the dropwise addition of 2M NaOH. The aqueous layer was then extracted with EtOAc (2 × 10 ml). The organic layer was then dried over anhydrous $MgSO_4$, filtered and evaporated *in vacuo* to give the title compound without the requirement of flash chromatography (0.051 g, 0.10 mmol, 97%). ^{1}H−NMR (400 MHz; $CDCl_3$): δ 7.47-7.45 (m, 2H), 7.33-7.31 (m, 3H), 7.03 (d, *J* = 8.5 Hz, 2H), 6.67 (d, *J* = 8.9 Hz, 2H), 5.76 (s, 1H), 4.37 (d, *J* = 6.6 Hz, 1H), 2.92 (s, 6H), 2.79-2.72 (m, 1H),

2.58-2.29 (m, 10H), 2.06-2.00 (m, 2H), 1.85-1.77 (m, 2H), 1.51-1.37 (m, 2H), 0.62 (s, 3H). ^{13}C−NMR (101 MHz; $CDCl_3$): δ 199.8, 157.0, 148.7, 146.7, 132.2, 131.8, 129.3, 128.45, 128.41, 127.6, 122.93, 122.84, 112.9, 92.5, 86.5, 80.5, 50.3, 47.5, 40.7, 39.7, 39.32, 39.12, 39.08, 36.9, 31.2, 27.5, 25.9, 23.6, 13.9. LCMS: 492.0 $[M+H]^+$

(8*S*,11*R*,13*S*,14*S*,17*S*)-11-(4-(dimethylamino)phenyl)-17-hydroxy-17-((4-methoxyphenyl)ethynyl)-13-methyl-1,2,6,7,8,11,12,13,14,15,16,17-dodecahydro-3*H*-cyclopenta[*a*]phenanthren-3-one (39)

37 (0.031 g, 0.05 mmol) was dissolved in 70% AcOH (2 ml) and heated to 50°C for 2 h, followed by diluted with H_2O and neutralization to pH 7 by the dropwise addition of 2 M NaOH. The aqueous layer was then extracted with EtOAc (2 × 10 ml). The organic layer was then dried over anhydrous $MgSO_4$, filtered and evaporated *in vacuo* to give the title compound without the requirement of flash chromatography (0.021 g, 0.04 mmol, 72%). ^{1}H−NMR (400 MHz; $CDCl_3$): δ 7.39 (d, *J* = 8.9 Hz, 2H), 7.04 (d, *J* = 8.5 Hz, 2H), 6.85 (d, *J* = 8.9 Hz, 2H), 6.68 (d, *J* = 8.5 Hz, 2H), 5.75 (s, 1H), 4.37 (d, *J* = 6.8 Hz, 1H), 3.81 (s, 3H), 2.92 (s, 6H), 2.79-2.73 (m, 1H), 2.59-2.56 (m, 2H), 2.52-2.29 (m, 7H), 2.09-2.00 (m, 3H), 1.80 (dt, *J* = 13.4, 6.4 Hz, 2H), 1.49-1.38 (m, 2H), 0.61 (s, 3H). ^{13}C−NMR (101 MHz; $CDCl_3$): δ 199.7, 159.8, 157.0, 146.7, 133.3, 129.3, 127.7, 122.9, 115.0, 114.1, 112.98, 112.95, 91.0, 86.5, 80.6, 55.5, 50.3, 47.5, 40.8, 39.8, 39.4, 39.2, 37.0, 31.3, 27.6, 26.0, 23.6, 13.9. LCMS: 522.0 $[M+H]^+$

(5*R*,8*S*,11*R*,13*S*,14*S*,17*S*)-13-methyl-11-phenyl-17-((trimethylsilyl)ethynyl)-1,2,6,7,8,11,12,13,14,15,16,17-dodecahydrospiro[cyclopenta[*a*]phenanthrene-3,2'-[1,3]dioxolane]-5,17(4*H*)-diol (45)

Magnesium Turnings (0.13 g, 5.5 mmol) and a crystal of iodine were spot-heated with a heat gun in anhydrous THF (3 ml). The temperature of the solution was maintained at ~50°C for 1 h.

After activation of the magnesium surface the solution was allowed to cool to rt. 4-bromobenzene (0.79 g, 5.04 mmol) was added dropwise in THF (4 ml) and following the complete addition the reaction was heated to 50°C for 1 h. **3** (0.5 g, 1.51 mmol) dissolved in anhydrous THF (4 ml) was cooled to 0°C and $CuCl_{(s)}$ (0.023 g, 0.23 mmol) was added and the reaction was stirred for 30 min. The Grignard solution was then added to this mixture dropwise at 0°C for 1 h, then the reaction was allowed to heat to rt. The reaction was allowed to proceed overnight and then reduced *in vacuo*. The crude mixture was then loaded onto a silica column and flash chromatography was conducted. Eluent used Pet. Spirits: EtOAc (3: 1) delivered the diketal intermediate **40** (0.18 g, 0.44 mmol, 29%) and was carried to the next step. Trimethylsilylacetylene (104 µl, 0.65 mmol) was dissolved in anhydrous THF (4 ml) and cooled to -78°C. To the solution 2.0 M *n*BuLi (366 µl) was added drop wise and following the complete addition the solution was allowed to stir for 1 h. After 1 h **40** (0.072 g, 0.16 mmol) in THF (1 mL) was added drop wise and the reaction was allowed to warm to rt and was stirred for 18 h. Upon completion the reaction was quenched with $NH_4Cl_{(aq)}$ and the crude mixture was extracted with EtOAc (2 × 10 ml). The organic fractions were collected, combined and reduced *in vacuo.* Crude material was loaded on silica column and flash chromatography using the eluent toluene: acetone (10: 1) gave the title compound (0.055 g, 0.11 mmol, 68%). ^{1}H−NMR (400 MHz; $CDCl_3$): δ 7.23-7.20 (m, 4H), 7.12-7.10 (m, 1H), 4.42 (s, 1H), 4.34-4.32 (m, 1H), 4.04-3.89 (m, 5H), 2.44-1.18 (m, 18H), 0.48-0.43 (m, 3H), 0.20 (s, 9H). ^{13}C−NMR (101 MHz; $CDCl_3$): δ 147.3, 134.4, 134.2, 128.2, 127.4, 125.4, 109.07, 108.89, 90.9, 80.6, 70.3, 64.8, 64.2, 49.7, 47.6, 46.8, 39.78, 39.59, 39.44, 39.32, 38.6, 35.1, 24.3, 24.0, 23.4, 13.6, 0.3. **LCMS: FAILED**

(5R,8S,13S,14S,17S)-11-(4-methoxyphenyl)-13-methyl-17-((trimethylsilyl)ethynyl)-1,2,6,7,8,11,12,13,14,15,16,17-dodecahydrospiro[cyclopenta[a]phenanthrene-3,2'-[1,3]dioxolane]-5,17(4H)-diol (46)

Magnesium Turnings (0.053 g, 2.22 mmol) and a crystal of iodine were spot-heated with a heat gun in anhydrous THF (3 ml). The temperature of the solution was maintained at ~50°C for 1 h. After activation of the magnesium surface the solution was allowed to cool to rt. 4-bromoanisole (0.38 g, 2.02 mmol) was added dropwise in THF (4 ml) and following the complete addition the reaction was heated to 50°C for 1 h. **3** (0.20 g, 0.61 mmol) dissolved in anhydrous THF (4 ml) was cooled to 0°C and $CuCl_{(s)}$ (0.009 g, 0.09 mmol) was added and the reaction was stirred for 30 min. The Grignard solution was then added to this mixture dropwise at 0°C for 1 h, then the reaction was allowed to heat to rt. The reaction was allowed to proceed overnight and then reduced *in vacuo*. The crude mixture was purified using flash chromatography eluent $CHCl_3$: MeOH (95: 5) to give **41** (0.13 g, 0.29 mmol, 47%). ^1H−NMR (400 MHz; $CDCl_3$): δ 7.12 (d, *J* = 8.2 Hz, 2H), 6.78 (d, *J* = 8.9 Hz, 2H), 4.36 (d, *J* = 1.0 Hz, 1H), 4.27 (d, *J* = 7.0 Hz, 1H), 4.03-3.90 (m, 4H), 3.77 (s, 3H), 2.46-2.39 (m, 3H), 2.37-2.29 (m, 2H), 2.06-2.00 (m, 4H), 1.88-1.75 (m, 3H), 1.62-1.51 (m, 5H), 1.29-1.21 (m, 1H), 0.49 (s, 3H). ^{13}C−NMR (101 MHz; CDCl3): δ 220.1, 157.5, 138.2, 135.2, 133.9, 128.1, 113.8, 108.8, 70.1, 64.8, 64.2, 55.3, 50.8, 47.63, 47.52, 38.7, 38.1, 37.90, 37.84, 35.7, 35.2, 23.54, 23.42, 22.3, 14.4. LCMS: 421.0 $[M-17]^+$ Trimethylsilylacetylene (115 µl, 0.72 mmol) was dissolved in anhydrous THF (4 ml) and cooled to -78°C. To the solution 2.0 M *n*BuLi (407 µl) was added drop wise and following the complete addition the solution was allowed to stir for 1 h. After 1 h **41** (0.079 g, 0.18 mmol) in THF (1 ml) was added drop wise and the reaction was allowed to warm to rt and was stirred for 18 h. Upon completion the reaction was quenched with $NH_4Cl_{(aq)}$ and the crude mixture was extracted with EtOAc (2 × 10 ml). The organic fractions were collected, combined and reduced *in vacuo*. Crude material was loaded on silica column and flash chromatography using the eluent toluene: acetone (10: 1) gave the title compound (0.007 g, 0.013 mmol, 7%). ^1H−NMR (400 MHz; $CDCl_3$): δ 7.13-7.10 (m, 2H), 6.80-6.77 (m, 2H), 4.41-4.40 (m, 1H), 4.28-4.26 (m, 1H), 4.04-3.90 (m, 4H), 3.78 (s, 3H), 2.42-1.22 (m, 19H), 0.46 (s, 3H), 0.19 (s, 9H). ^{13}C−NMR (101 MHz; CDCl3): δ

157.3, 139.1, 134.5, 134.2, 128.3, 113.6, 108.9, 90.9, 80.6, 70.3, 64.8, 64.2, 55.3, 49.7, 47.7, 46.8, 39.53, 39.41, 39.33, 39.0, 38.6, 35.2, 24.3, 24.0, 23.4, 13.7, 0.3.

LCMS FAILED

(5*R*,8*S*,11*R*,13*S*,14*S*,17*S*)-11-([1,1'-biphenyl]-4-yl)-13-methyl-17-((trimethylsilyl)ethynyl)-1,2,6,7,8,11,12,13,14,15,16,17-dodecahydrospiro[cyclopenta[*a*]phenanthrene-3,2'-[1,3]dioxolane]-5,17(4*H*)-diol (48)

Magnesium Turnings (0.042 g, 1.73 mmol) and a crystal of iodine were spot-heated with a heat gun in anhydrous THF (3 ml). The temperature of the solution was maintained at ~50°C for 1 h. After activation of the magnesium surface the solution was allowed to cool to rt. 4-bromobiphenyl (0.37 g, 1.59 mmol) was added dropwise in THF (4 ml) and following the complete addition the reaction was heated to 50°C for 1 h. **3** (0.16 g, 0.48 mmol) dissolved in anhydrous THF (4 ml) was cooled to 0°C and $CuCl_{(s)}$ (0.007 g, 0.072 mmol) was added and the reaction was stirred for 30 min. The Grignard solution was then added to this mixture dropwise at 0°C for 1 h, then the reaction was allowed to heat to rt. The reaction was allowed to proceed overnight and then reduced *in vacuo*. The crude mixture was then carried forward. Trimethylsilylacetylene (416 µl, 1.44 mmol) was dissolved in anhydrous THF (6 ml) and cooled to -78°C. To the solution 2.0 M *n*BuLi (810 µl) was added drop wise and following the complete addition the solution was allowed to stir for 1 h. After 1 h **43** (0.072 g, 0.16 mmol) in THF (1 mL) was added drop wise and the reaction was allowed to warm to rt and was stirred for 18 h. Upon completion the reaction was quenched with $NH_4Cl_{(aq)}$ and the crude mixture was extracted with EtOAc (2 × 10 ml). The organic fractions were collected, combined and reduced *in vacuo.* Crude material was loaded on silica column and flash chromatography using the eluent toluene: acetone (10: 1) gave the title compound (0.035 g, 0.06 mmol, 37%). ^{1}H–NMR (400 MHz; $CDCl_3$): δ 7.61-7.58 (m, 2H), 7.50-7.48 (m, 2H), 7.42 (dd, *J* = 8.2, 7.0 Hz, 2H), 7.33-7.28 (m,

3H), 4.43 (s, 1H), 4.37 (d, *J* = 7.3 Hz, 1H), 4.04-3.89 (m, 4H), 2.49-2.32 (m, 4H), 2.30-2.23 (m, 2H), 2.20-2.14 (m, 1H), 2.07-2.02 (m, 2H), 1.97-1.90 (m, 2H), 1.83-1.53 (m, 6H), 1.35-1.24 (m, 2H), 0.50 (s, 3H), 0.21 (s, 9H). ^{13}C−NMR (101 MHz; CDCl3): δ 146.4, 140.9, 138.1, 134.5, 134.1, 129.1, 128.8, 128.3, 127.8, 127.11, 126.95, 126.82, 125.4, 109.1, 108.9, 90.9, 80.6, 70.3, 64.8, 64.1, 49.7, 47.7, 46.9, 39.56, 39.39, 39.35, 38.6, 35.2, 24.3, 24.0, 23.5, 13.8, 0.3. **LCMS: FAILED**

(5*R*,8*S*,11*R*,13*S*,14*S*,17*S*)-11-(3-(dimethylamino)phenyl)-13-methyl-17-((trimethylsilyl)ethynyl)-1,2,6,7,8,11,12,13,14,15,16,17-dodecahydrospiro[cyclopenta[*a*]phenanthrene-3,2'-[1,3]dioxolane]-5,17(4*H*)-diol (49)

Magnesium Turnings (0.04 g, 1.47 mmol) and a crystal of iodine were spot-heated with a heat gun in anhydrous THF (3 ml). The temperature of the solution was maintained at ~50°C for 1 h. After activation of the magnesium surface the solution was allowed to cool to rt. 3-bromo-N,N-dimethylaniline (0.27 g, 1.34 mmol) was added drop wise in THF (3 ml) and following the complete addition the reaction was heated to 50°C for 1 h. Separately **3** (0.13 g, 0.4 mmol) dissolved in anhydrous THF (2 ml) was cooled to 0°C and $CuCl_{(s)}$ (0.006 g, 0.06 mmol) was added and the reaction was stirred for 30 min. The Grignard solution was then added to this mixture dropwise at 0°C for 1 h then the reaction was allowed to heat to rt. The reaction was allowed to proceed overnight and then reduced *in vacuo*. The crude mixture was then carried forward to the next step. Trimethylsilylacetylene (462 µl, 1.6 mmol) was dissolved in anhydrous THF (6 ml) and cooled to -78°C. To the solution 2.0 M *n*BuLi (900 µl) was added drop wise and following the complete addition the solution was allowed to stir for 1 h. After 1 h crude in THF (1 mL) was added drop wise and the reaction was allowed to warm to rt and was stirred for 18 h. Upon completion the reaction was quenched with $NH_4Cl_{(aq)}$ and the crude mixture was extracted with EtOAc (2 × 10 ml). The organic fractions were collected, combined and reduced *in vacuo.*

Crude material was loaded on silica column and flash chromatography using the eluent used Pet. Spirits: EtOAc (3: 1) delivered the title compound (0.021 g, 0.04 mmol, 10%). ^{1}H−NMR (400 MHz; $CDCl_3$): δ 7.10 (t, *J* = 7.9 Hz, 1H), 6.63-6.60 (m, 2H), 6.52 (dd, *J* = 8.1, 2.1 Hz, 1H), 4.42 (s, 1H), 4.29 (dd, *J* = 6.9, 0.2 Hz, 1H), 4.03-3.90 (m, 4H), 2.92 (s, 6H), 2.50-2.44 (m, 1H), 2.37-2.22 (m, 4H), 2.19-2.11 (m, 1H), 2.05-2.00 (m, 2H), 1.96-1.89 (m, 1H), 1.81-1.50 (m, 8H), 1.36-1.25 (m, 2H), 0.51 (s, 3H), 0.20 (s, 9H). ^{13}C−NMR (101 MHz; CDCl3): δ 150.6, 148.0, 134.8, 134.2, 128.7, 116.2, 112.1, 109.9, 109.2, 108.9, 90.8, 80.6, 70.4, 64.8, 64.2, 49.9, 47.8, 46.9, 40.9, 39.9, 39.7, 39.40, 39.22, 35.3, 24.2, 24.0, 23.4, 13.5, 0.3. LCMS: 532.4 $[M-17]^+$

(8S,13S,14S,17S)-17-hydroxy-13-methyl-11-phenyl-17-((trimethylsilyl)ethynyl)-1,2,6,7,8,11,12,13,14,15,16,17-dodecahydro-3H-cyclopenta[a]phenanthren-3-one (50)

45 (0.05 g, 0.1 mmol) was dissolved in 70% AcOH (2 ml) and heated to 50°C for 2 h, followed by dilution with H_2O and neutralization to pH 7 by dropwise addition of 2M NaOH. The aqueous layer was then extracted with CH_2Cl_2 (2 × 10 ml). The organic layer was then dried over anhydrous $MgSO_4$, filtered and evaporated *in vacuo* to give the title compound without the requirement of flash chromatography (0.048 g, 0.1 mmol, 99%). ^{1}H−NMR (400 MHz; $CDCl_3$): δ 7.29-7.25 (m, 2H), 7.16 (dd, *J* = 15.3, 7.5 Hz, 3H), 5.78 (s, 1H), 4.44 (d, *J* = 6.8 Hz, 1H), 2.79-2.73 (m, 1H), 2.63-2.58 (m, 2H), 2.51-1.33 (m, 14H), 0.50 (s, 3H), 0.19 (s, 9H). ^{13}C−NMR (101 MHz; $CDCl_3$): δ 199.6, 156.8, 145.8, 144.7, 129.7, 128.6, 127.1, 125.9, 123.1, 109.0, 91.1, 80.3, 50.1, 47.1, 40.6, 39.30, 39.17, 39.02, 37.0, 31.2, 27.5, 26.0, 23.5, 13.8, 0.2. LCMS: 445.0 $[M+H]^+$

(8S,13S,14S,17S)-17-hydroxy-11-(4-methoxyphenyl)-13-methyl-17-((trimethylsilyl)ethynyl)-1,2,6,7,8,11,12,13,14,15,16,17-dodecahydro-3H-cyclopenta[a]phenanthren-3-one (51)

46 (0.023 g, 0.004 mmol) was dissolved in 70% AcOH (2 ml) and heated to 50°C for 2 h, followed by dilution with H_2O and neutralization to pH 7 by dropwise addition of 2 M NaOH. The aqueous layer was then extracted with CH_2Cl_2 (2 × 10 ml). The organic layer was then dried over anhydrous $MgSO_4$, filtered and evaporated *in vacuo*. Crude material was purified by flash chromatography eluent toluene: acetone (7: 1) to give the title compound (0.001 g, 0.002 mmol, 52%**).** ^{1}H−NMR (400 MHz; $CDCl_3$): δ 7.10-7.07 (m, 2H), 6.82 (d, J = 8.9 Hz, 2H), 5.77 (d, J = 0.2 Hz, 1H), 4.40-4.38 (m, 1H), 3.78 (s, 3H), 2.80-2.73 (m, 1H), 2.61-2.57 (m, 2H), 2.49-2.24 (m, 5H), 2.05-1.93 (m, 3H), 1.83-1.67 (m, 3H), 0.90-0.80 (m, 3H), 0.53 (s, 3H), 0.19 (s, 9H). ^{13}C−NMR (101 MHz; $CDCl_3$): δ 199.2, 157.8, 156.7, 146.1, 136.5, 129.6, 128.1, 123.2, 114.1, 55.4, 50.2, 47.1, 39.9, 39.28, 39.21, 39.11, 37.1, 31.2, 27.5, 26.0, 23.6, 13.9, 0.2.

LCMS FAILED

(8*S*,11*R*,13*S*,14*S*,17*S*)-17-hydroxy-11-(4-hydroxyphenyl)-13-methyl-17-((trimethylsilyl)ethynyl)-1,2,6,7,8,11,12,13,14,15,16,17-dodecahydro-3*H*-cyclopenta[*a*]phenanthren-3-one (52)

Magnesium Turnings (0.05 g, 2.2 mmol) and a crystal of iodine were spot-heated with a heat gun in anhydrous THF (3 ml). The temperature of the solution was maintained at ~50°C for 1 h. After activation of the magnesium surface the solution was allowed to cool to rt. 2-(4-Bromophenoxy)tetrahydro-2*H*-pyran (0.52 g, 2.02 mmol) was added dropwise in THF (2 ml) and following the complete addition the reaction was heated to 50°C for 1 h. Separately **3** (0.2 g, 0.6 mmol) dissolved in anhydrous THF (10 ml) was cooled to 0°C and $CuCl_{(s)}$ (0.01 g, 0.09 mmol) was added and the reaction was stirred for 30 min. The Grignard solution was then added to this mixture dropwise at 0°C for 1 h, then the reaction was allowed to heat to rt. The reaction was allowed to proceed overnight and then reduced *in vacuo*. The crude mixture was then loaded onto a silica column and flash chromatography was conducted. Eluent used Pet. Spirits: EtOAc

(2: 1) delivered intermediate **42** (0.21 g, 0.40 mmol, 67%) and was carried to the next step. ^{1}H−NMR (400 MHz; $CDCl_3$): δ 7.09 (d, *J* = 8.8 Hz, 2H), 6.91 (dd, *J* = 8.8, 1.2 Hz, 2H), 5.31 (dt, *J* = 12.1, 3.4 Hz, 1H), 4.35 (d, *J* = 3.8 Hz, 1H), 4.26-4.24 (m, 1H), 4.01-3.87 (m, 5H), 3.60-3.56 (m, 1H), 2.44-2.27 (m, 5H), 2.08-1.96 (m, 5H), 1.85-1.73 (m, 5H), 1.67-1.49 (m, 8H), 1.26-1.23 (m, 1H), 0.48 (s, 3H). ^{13}C−NMR (101 MHz; $CDCl_3$): δ 220.04, 219.98, 155.14, 154.96, 139.07, 138.99, 135.1, 133.8, 128.00, 127.95, 116.4, 116.1, 108.7, 96.9, 96.6, 70.1, 64.7, 64.1, 62.42, 62.37, 50.77, 50.72, 47.58, 47.57, 47.45, 47.41, 38.7, 38.06, 38.02, 37.76, 37.70, 35.65, 35.64, 35.10, 35.09, 30.59, 30.53, 25.29, 25.27, 23.47, 23.34, 22.2, 19.1, 14.38, 14.35. LCMS: 490.2 $[M+H]^+$

Trimethylsilylacetylene (178 µl, 1.1 mmol) was added to anhydrous THF (4 ml) and cooled to -78°C. To the solution 2 M *n*BuLi (625 µl) was added drop wise and following the complete addition the solution was allowed to stir for 1 h. After 1 h **42** (0.14 g, 0.28 mmol) in THF (1 mL) was added drop wise and the reaction was allowed to warm to rt and was stirred for 18 h. Upon completion the reaction was quenched with $NH_4Cl_{(aq)}$ and the crude mixture was extracted with EtOAc (2 × 10 ml). The organic fractions were collected, combined and reduced *in vacuo.* Crude material was carried to the next step due to instability issues. **47** (0.06 g, 0.1 mmol) was dissolved in 70% AcOH (2 mL) and heated to 50°C for 2 h. The crude reaction was then diluted with H_2O and neutralized to pH 7 by the drop wise addition of 2 M NaOH. The aqueous layer was then extracted with CH_2Cl_2 (2 × 10 ml). The organic layer was then dried over anhydrous $MgSO_4$, filtered and evaporated *in vacuo.* Crude product was isolated using flash chromatography using eluent toluene: acetone (10: 1) to give the title compound (0.03 g, 0.06 mmol, 60%). ^{1}H−NMR (400 MHz; $CDCl_3$): δ 7.00 (d, *J* = 8.5 Hz, 2H), 6.74 (d, *J* = 8.7 Hz, 2H), 6.08-6.00 (m, 1H), 5.79 (s, 1H), 4.35 (d, *J* = 6.9 Hz, 1H), 2.77-2.70 (m, 1H), 2.60-2.58 (m, 2H), 2.49-2.41 (m, 2H), 2.38-2.24 (m, 5H), 2.05-1.95 (m, 3H), 1.80-1.66 (m, 2H), 1.52-1.42 (m, 1H), 1.41-1.33 (m, 1H), 0.52 (s, 3H), 0.18 (s, 9H). ^{13}C−NMR (101 MHz; $CDCl_3$): δ 200.4, 157.5,

154.05, 146.8, 136.2, 129.5, 128.1, 122.9, 115.6, 108.9, 91.2, 80.4, 50.1, 47.0, 39.9, 39.29, 39.19, 39.01, 36.9, 31.2, 27.4, 25.9, 23.6, 13.8, 0.2. LCMS: 461.0 $[M+H]^+$

(8*S*,11*R*,13*S*,14*S*,17*S*)-11-([1,1'-biphenyl]-4-yl)-17-hydroxy-13-methyl-17-((trimethylsilyl)ethynyl)-1,2,6,7,8,11,12,13,14,15,16,17-dodecahydro-3*H*-cyclopenta[*a*]phenanthren-3-one (53)

48 (0.10 g, 0.18 mmol) was dissolved in 70% AcOH (2 ml) and heated to 50°C for 2 h, followed by dilution with H_2O and neutralization to pH 7 by the dropwise addition of 2 M NaOH. The aqueous layer was then extracted with CH_2Cl_2 (2 × 10 ml). The organic layer was then dried over anhydrous $MgSO_4$, filtered and evaporated *in vacuo*. Crude product was isolated using flash chromatography using eluent toluene: acetone (11: 1) to give the title compound (0.06 g, 0.11 mmol, 60%). ^{1}H–NMR (400 MHz; $CDCl_3$): δ 7.60-7.57 (m, 2H), 7.53-7.51 (m, 2H), 7.45-7.41 (m, 2H), 7.35-7.33 (m, 1H), 7.26-7.24 (m, 2H), 5.80 (s, 1H), 4.49-4.48 (m, 1H), 2.82-2.77 (m, 1H), 2.64-2.60 (m, 2H), 2.54-1.36 (m, 14H), 0.56 (s, 3H), 0.20 (s, 9H). ^{13}C–NMR (101 MHz; $CDCl_3$): δ 199.6, 156.7, 145.8, 143.8, 140.7, 138.7, 129.8, 128.9, 127.5, 127.32, 127.29, 127.0, 123.2, 109.0, 91.2, 80.3, 50.2, 47.2, 40.4, 39.31, 39.18, 39.10, 37.1, 31.2, 27.5, 26.1, 23.6, 14.0, 0.2. LCMS: 520.4 $[M]^+$

(8*S*,11*R*,13*S*,14*S*,17*S*)-11-(3-(dimethylamino)phenyl)-17-hydroxy-13-methyl-17-((trimethylsilyl)ethynyl)-1,2,6,7,8,11,12,13,14,15,16,17-dodecahydro-3*H*-cyclopenta[*a*]phenanthren-3-one (54)

49 (0.01 g, 0.018 mmol) was dissolved in 70% AcOH (2 ml) and heated to 50°C for 2 h, followed by dilution with H_2O and neutralization to pH 7 by the dropwise addition of 2 M NaOH. The aqueous layer was then extracted with CH_2Cl_2 (2 × 10 ml). The organic layer was then dried over anhydrous $MgSO_4$, filtered and evaporated *in vacuo*. Crude product was isolated

using flash chromatography using eluent toluene: acetone (10: 1) to give the title compound (0.007 g, 0.014 mmol, 81%). ^{1}H−NMR (400 MHz; $CDCl_3$): δ 7.12 (t, *J* = 7.9 Hz, 1H), 6.58-6.53 (m, 3H), 5.77 (s, 1H), 4.41-4.39 (m, 1H), 2.92 (s, 6H), 2.81-2.74 (m, 1H), 2.59-2.55 (m, 2H), 2.51-1.34 (m, 14H), 0.59 (s, 3H), 0.19 (s, 9H). ^{13}C−NMR (101 MHz; $CDCl_3$): δ 199.8, 156.9, 150.9, 146.5, 145.4, 129.6, 129.1, 128.4, 123.0, 115.6, 111.7, 110.3, 109.0, 91.1, 80.4, 50.3, 47.2, 40.86, 40.78, 39.5, 39.0, 37.1, 31.2, 26.1, 23.5, 13.7, 0.2. LCMS: 488.4 $[M+H]^+$
